# Numerical simulation and parameter optimization of micromixer device using fuzzy logic technique

**DOI:** 10.1039/d2ra07992e

**Published:** 2023-02-02

**Authors:** Karthikeyan K, Senthil Kumar Kandasamy, Saravanan P, Abdullah Alodhayb

**Affiliations:** a Department of Electronics and Communication Engineering, M.Kumarasamy College of Engineering Karur Tamil Nadu India karthimems@gmail.com; b Department of Electronics and Communication Engineering, Kongu Engineering College Erode Tamil Nadu India senthilkumar6k@gmail.com; c Department of Self Development Skills, CFY Deanship, King Saud University Riyadh Saudi Arabia psaravanan.c@ksu.edu.sa; d Department of Physics and Astronomy, College of Science, King Saud University Riyadh Saudi Arabia aalodhayb@ksu.edu.sa

## Abstract

The objective of this study is the design, simulation, and performance optimization of a micromixer device using the three input parameters of device structure, flow rate and diffusion coefficient of gold nanoparticles while the output parameters are concentration, velocity, pressure and time domain analysis. Each input parameter in the microfluidic chip influences the system output. The data were gathered through extensive study in order to optimize the diffusion control. The fuzzy logic approach is used to optimize the performance of the device with respect to the input parameters. In this study, we have chosen three different flow rates of 1, 5, and 10 μL min^−1^, three different diffusion coefficient values of low, average and high diffusivity gold nanofluids (15.3 e^−12^, 15.3 e^−11^, 15.3 e^−10^ m^2^ s^−1^) which are used in three different shapes of micromixer device, Y-shaped straight channel micromixer, herringbone-shaped micromixer, and herringbone shape with obstacles micromixer, and we measured the output performance, such as mixing efficiency, pressure drop, concentration across the microchannel and time domain. The data were obtained by fuzzy logic analysis and it was found that the herringbone shape with obstacles micromixer shows 100% mixing efficiency within a short duration of 5000 μm, and complete mixing was achieved within 10 seconds with a low pressure drop of 128 Pa.

## Abstract 2

Simulated result of Y-shaped herringbone serpentine channel micromixer with obstacles.

## Graphical abstract

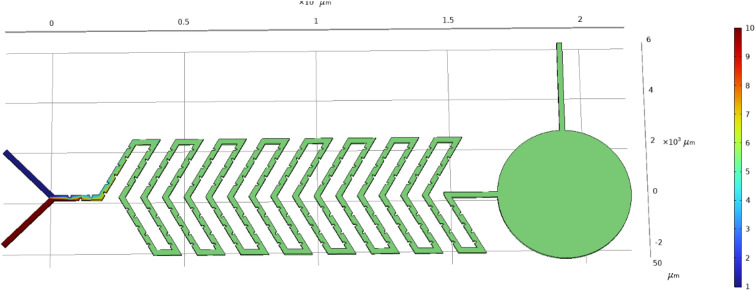

## 1 Introduction

A microfluidic device provides a powerful tool for lab on a chip (LOC) applications, such as sensing,^1^ DAN amplification,^2^ synthesis of nanoparticles^3^ and blood cell separation. A microfluidic device offers a portable diagnostic device for point-of-care (POC) applications. There are several components in LOC and POC systems, such as microwells, microchannels, reservoirs, mixers, reactors, pumps and valves. A micromixer is an important component, which is used to mix fluids in the range of micro–nano–pico liters. In microfluidics for lab-on-a-chip applications, mixing performance remains a major challenge because fluid flow is generally laminar. Generally, micromixers are classified into two types: active and passive micromixer devices. When an external force is required to mix the fluids, we can call it an active micromixer. Different types of fields are used in an active micromixer, such as magnetic,^4,5^ radio frequency,^6^ electroosmotic^7,8^ and surface acoustic wave.^9–11^ Similarly, a passive micromixer device can have different physiological structures, such as Y-type,^12^ T-shape,^13^ grooves/obstacles,^14^ split–recombine,^15^ serpentine^16^ and herringbone type structures.^17^ The construction of an active micromixer has greater complexity than a passive micromixer. Unfortunately, the driver voltage is too high, so the cost does not match the power. In addition, they are very difficult to make, so their uses are limited. At the same time, passive micromixers are simple, inexpensive devices and do not require an external field to induce mixing efficiency. Additionally, they can be improved by modifying the microchannel structure, which helps improve mixing.^18,19^

S. Camarri *et al.*^20^ studied the engulfment regime of a T-type and T-joint micromixer device and reported the mixing efficiency and pressure drop. They reported that the configuration of a CA1 or CA2 device shows better efficiency than an isolated T-micromixer. When comparing the pressure drop between two the different configurations of CA1 and CA2 devices, the configuration of the CA1 device shows a lower pressure drop compared to CA2. X. Zhan *et al.*^21^ designed a T-type micromixer with three different structural shapes: elliptical, rectangular and triangular shaped microchannels. Better mixing efficiency was found while using an elliptical cross-sectional microchannel. Z. Wu *et al.*^22^ reported the design and numerical simulation of a three-dimensional T-shaped passive micromixer with three different obstacles: square, triangular and cylindrical. They reported a mixing efficiency of 96% while using the triangular obstacles with Re = 100 and 18 kPa pressure drop. E. Tripathi *et al.*^23^ designed and reported a spiral shaped micromixer which was investigated for a wide range of Reynolds numbers between 0.1 and 100. Better mixing was observed within the range of Reynolds number from 0.1 to 50. E. Nady *et al.*^24^ studied the two inlets and two outlets of a Y-type passive micromixer with circular obstacles and tall walls. The total length of the device is 14 mm, the width of the channel is 200 μm, the thickness of the wall is 30 μm and the gaps between the walls are 170 μm and 70 μm. Better mixing was achieved in a short distance while using a circular channel with high number of tall wall structure and it act as an obstacles. Y. Liao *et al.*^25^ reported a passive micromixer device with staggered herring bone structure and split–recombination microchannel. The mixing efficiency was analysed using a wide range of flow rates, 1–12 μL min^−1^, and Reynolds numbers 3.3–40 and they achieved 98% mixing efficiency at 4.5–78 milliseconds. M. Ripoll *et al.*^26^ designed a Y-type ring shaped micromixer which was used to produce lipid nanoparticles through the mixing of lipids and biomolecules. They reported the mixing performance to be linked with the characteristics of the lipid nanoparticles. O. Ulkir *et al.*^27^ designed a T-shaped laminar diffusion-based micromixer with two inlets and two outlets. The mixing efficiency was studied using the diffusion coefficient 5 e^−11^ m^2^ s^−1^ and inlet flow rate of 15 e^−15^ m^3^ s^−1^. For the output value of the system, the velocity was 0.09 mm s^−1^, the pressure was 2 Pa and the concentration was 0.45 mol m^−3^. Karthikeyan *et al.*^28^ reported a Y-type herringbone shaped micromixer for mercury ion detection in water. They studied the pressure level and mixing efficiency of the device at different locations.

V. Vijayanandh *et al.*^29^ reported a T-type micromixer with different shapes of ridges, such as triangular, square and curved. The mixing efficiency of the device was optimised using different shapes of micromixers and they reported that the best mixing efficiency was achieved while using a micromixer with triangular ridges. Karthikeyan *et al.*^16^ studied the different shapes of micromixers such as a Y-type straight channel micromixer, and a serpentine shape micromixer with or without grooves. They reported the mixing efficiency and pressure drop. The best mixing efficiency was achieved with a short length while using a micromixer with grooves.

S. Hossain *et al.*^30^ reported a serpentine micromixer with the crossing of two layers. They studied a mixing efficiency of 96% at low Reynolds numbers from 0.2 to 10 and low pressure drop. X. Dong *et al.*^31^ designed a T-shaped micromixer for a non-Newtonian fluid. They studied a mixing efficiency of 93.84% at Re = 0.24 while using a non-Newtonian fluid and 93.90% mixing efficiency at Re = 8 while using a Newtonian fluid.

Karthikeyan *et al.*^12^ designed a Y-shaped micromixer with rectangular and triangular shaped obstacles to mix fluids with very low diffusivity. The mixing efficiency observed for the triangular shaped micromixer shows 100% mixing efficiency compared with other micromixers with rectangular shaped obstacles at a flow rate corresponding to the Reynolds number (Re) of 25. I. Ertugrul *et al.*^32^ reported a microfluidic device for platelet separation using the fuzzy logic technique.

M. Hejazian *et al.*^33^ reported a straight and serpentine shaped micromixer. The mixing efficiency of the device was studied using fluorescence intensity profiles with different flow rates of 20, 100 and 200 μL min^−1^. A. Usefian *et al.*^34^ designed a Y-shaped convergence and divergence-based micromixer for low flow rate applications. S. R. Bazaz *et al.*^35^ developed three different shapes of passive hybrid planar micromixer with repetitive obstacles, such as teardrop, nozzle, ellipse, pillar and tesla shaped obstructions inside the mixing zone. R. A. Taheri *et al.*^36^ reported a three-dimensional micromixer with split and recombine microchannel. They reported 96% mixing efficiency at a Reynolds number of 0.1, 90% mixing efficiency at a Reynolds number of 1 and 67% mixing efficiency at a Reynolds number of 10.

In this paper, we propose three dissimilar structures of micromixer: a Y-shaped straight channel micromixer (SCM), a herringbone serpentine channel micromixer (HSM), and a herringbone serpentine channel micromixer with obstacles (HSOM). The characteristic performance of the devices is discussed using the three input parameters of device structure, flow rate and diffusion coefficient, while the outputs are concentration, mixing efficiency, velocity, pressure and time domain analysis.

## 2 Design of micromixer

### 2.1 Y-shaped straight channel micromixer

**Fig. 1:**
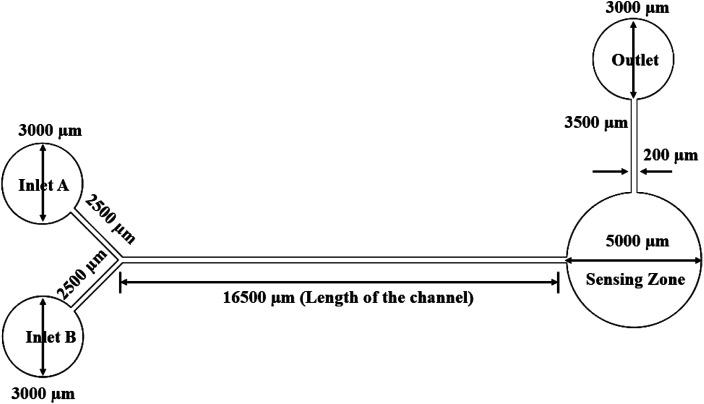
Y-shaped straight channel micromixer.

A Y-shaped micromixer is one of the simplest models used to mix two liquids A and B. This micromixer contains a long straight channel of around 16 500 μm (16 mm) with two inlets each of about 2500 μm in length. The width of the microchannel is 200 μm and the diameter of the inlet and outlet reservoirs is 3000 μm. This device has a sensing zone diameter of 5000 μm. The structure of the Y-shaped micromixer with a straight channel and its dimensions are shown in Fig. 1.

### 2.2 Y-shaped herringbone serpentine channel micromixer

**Fig. 2:**
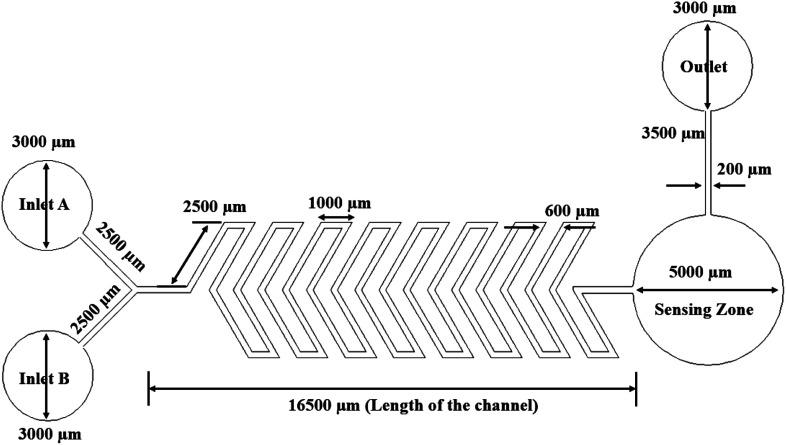
Y-shaped herringbone serpentine channel micromixer.

The Y-shaped herringbone serpentine channel micromixer contains many sharp bends in the microchannel. The width and overall length of the microchannel are around 200 μm and 16 mm (*i.e. x*-axis 16 mm), respectively, with two inlets each of 2500 μm length. The space between two bends is 200 μm. This device has a sensing zone diameter of 5000 μm. The structure of the Y-shaped herringbone serpentine channel micromixer and its dimensions are shown in Fig. 2.

### 2.3 Y-shaped herringbone serpentine channel micromixer with obstacles

**Fig. 3:**
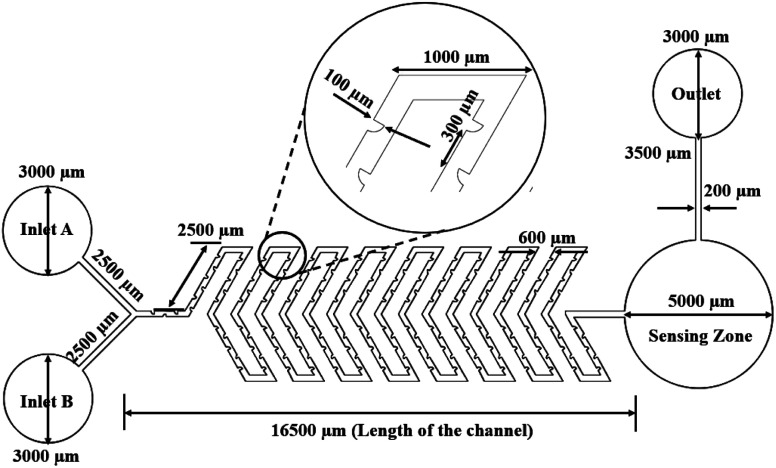
Y-shaped herringbone serpentine channel micromixer with obstacles.

The structure of the Y-shaped herringbone serpentine channel micromixer with obstacles and its dimensions are shown in Fig. 3. The obstacles are of quadrant shape, as shown in the insert to Fig. 3. The quadrant shaped obstacles have a smooth curved edge at the fluid inlet, which provides smooth fluid flow, and a vertical edge at the other end, which improves fluid interaction. The obstacles improve the mixing efficiency over a short length. The grooves are kept at a spacing of 100 μm and there are 164 obstacles over the whole mixing length of 16 mm with two inlets each of 2500 μm length and an inlet port diameter of 3000 μm. This device has a sensing zone diameter of 5000 μm.

## 3 Simulations of micromixers

Simulations were carried out with the Numerical Multiphysics CAD tool. The structures were drawn using the design values given in the previous section.

### 3.1 Analytical expressions for micromixing

The flow of an incompressible Newtonian liquid in a micromixer can be described by the Navier–Stokes equation and continuity equation, as shown in eqn (1) and (2), respectively.1
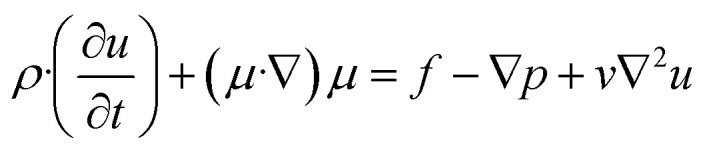

2 ∇·*u* = 0

where *ρ* is the fluid density, *u* is the flow velocity, *v* is the dynamic viscosity of the fluid, *p* is the fluid pressure, and *f* is the body force.

The species transport in the systems can be described by the convection diffusion equation, as shown in eqn (3),3
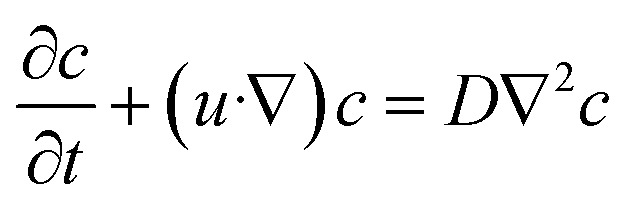
where *c* and *D* are the concentration and diffusion constant of the species. The term “pressure drop” refers to the drop in pressure across the geometry of any device. *i.e.* the difference between inlet pressure and outlet pressure.

Mathematically it can be represented as,

4 Δ*P* = *P*_inlet_ − *P*_outlet_

The mixing efficiency (*M*) of the micromixer can be calculated using the following formula,5
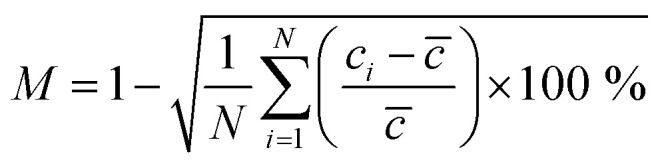
where *N* is the total number of sampling points across the cross-section in the channel, *c*_*i*_ is the normalized concentration of the fluid at each cross-section of the device, and *c̄* is the average concentration of the fluid in the inlets. In accordance with eqn (5), the mixing efficiency, *M* = 0% indicates the completely unmixed state of the species, and *M* = 100% indicates the completely mixed state. An efficiency of mixing between about 80 and 100% is suitable for mixing applications.^37,38^

### 3.2 Analysis of micromixer

#### 3.2.1 Simulated micromixer device

**Fig. 4:**
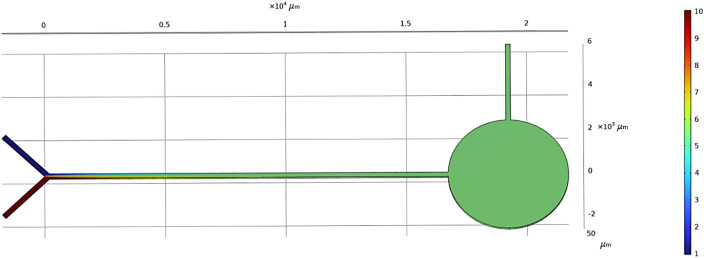
Simulated result of Y-shaped straight channel micromixer.

**Fig. 5:**
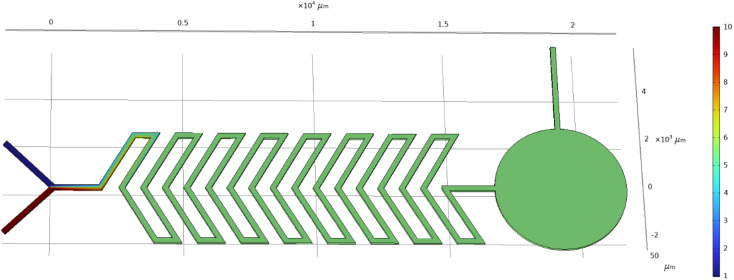
Simulated result of Y-shaped herringbone serpentine channel micromixer.

**Fig. 6:**
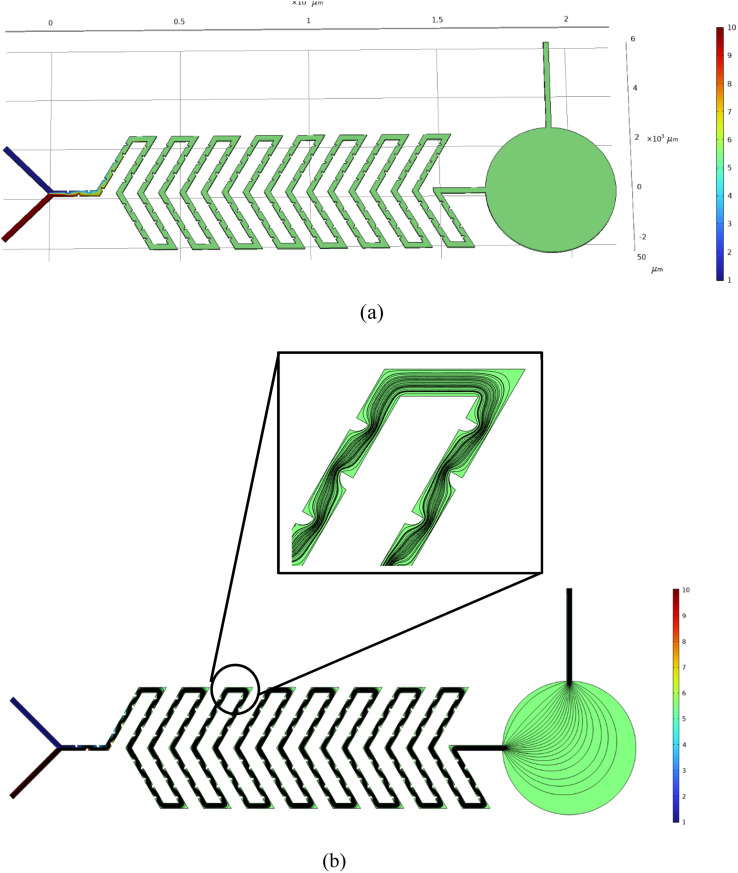
Simulated result of Y-shaped herringbone serpentine channel micromixer with obstacles: (a) concentration field and (b) streamline distribution at 5000 μm.

We look at the micromixer model processes of a microfluidic device for controlled mixing by diffusion. The device brings two different laminar streams into contact for a controlled time. The contact surface is well defined, and by controlling the flow rate, it is possible to control the number of species transferred from one stream to another by diffusion. Diagrams of the microfluidic-based micromixer devices to be analyzed, each with two inputs and an output, are shown in Fig. 4, 5 and 6.

Fig. 4 shows the concentration level across the device of a Y-shaped straight channel micromixer. This device has two inlets in a Y-shape with a straight channel acting as a mixing zone followed by a sensing zone and outlet. Fig. 4 shows a simulation study of “Test case 1”, which is presented in Table 1. Fig. 5 shows the concentration level across the device of a Y-shaped herringbone serpentine channel micromixer. This device has two inlets in a Y-shape with a herringbone serpentine channel acting as a mixing zone followed by a sensing zone and outlet. Fig. 5 shows the simulation study of “Test case 10”, which is presented in Table 1. Fig. 6 shows the concentration level across the device of a Y-shaped herringbone serpentine channel micromixer with obstacles. This device has two inlets in a Y-shape along with a herringbone serpentine channel with obstacles acting as a mixing zone followed by a sensing zone and outlet. Fig. 6 shows the simulation study of “Test case 19”, which is presented in Table 1, Fig. 4, 5, and 6 show the mixing concentration profile across the device with different structures and the same input parameters. In Fig. 4, the fluid flow is laminar due to the microchannel and it requires a greater length of microchannel to achieve complete mixing: in this case complete mixing was achieved at 17 500 μm. Therefore, Fig. 5 shows a Y-shaped herringbone serpentine channel with sharp edges for better mixing and mixing was achieved at 7500 μm. Then Fig. 6(a) and (b) present a Y-shaped herringbone serpentine channel with obstacles to achieve complete mixing within short length of under 5000 μm.

**Table 1:** Fuzzy logic test case table

Test case	Inputs			Outputs		
	Device structure	Flow rate	Diffusion coefficient	Velocity (mm s^−1^)	Pressure (Pa)	Concentration (mol m^−3^)
TC1	Straight channel	1 μL min^−1^	15.3 e^−10^	Minimum	Minimum	Maximum
TC2		1 μL min^−1^	15.3 e^−11^	Average	Average	Average
TC3		1 μL min^−1^	15.3 e^−12^	Maximum	Maximum	Minimum
TC4		5 μL min^−1^	15.3 e^−10^	Minimum	Minimum	Maximum
TC5		5 μL min^−1^	15.3 e^−11^	Average	Average	Average
TC6		5 μL min^−1^	15.3 e^−12^	Maximum	Maximum	Minimum
TC7		10 μL min^−1^	15.3 e^−10^	Minimum	Minimum	Maximum
TC8		10 μL min^−1^	15.3 e^−11^	Average	Average	Average
TC9		10 μL min^−1^	15.3 e^−12^	Maximum	Maximum	Minimum
TC10	Herring bone serpentine channel	1 μL min^−1^	15.3 e^−10^	Minimum	Minimum	Maximum
TC11		1 μL min^−1^	15.3 e^−11^	Average	Average	Average
TC12		1 μL min^−1^	15.3 e^−12^	Maximum	Maximum	Minimum
TC13		5 μL min^−1^	15.3 e^−10^	Minimum	Minimum	Maximum
TC14		5 μL min^−1^	15.3 e^−11^	Average	Average	Average
TC15		5 μL min^−1^	15.3 e^−12^	Maximum	Maximum	Minimum
TC16		10 μL min^−1^	15.3 e^−10^	Minimum	Minimum	Maximum
TC17		10 μL min^−1^	15.3 e^−11^	Average	Average	Average
TC18		10 μL min^−1^	15.3 e^−12^	Maximum	Maximum	Minimum
TC19	Herring bone serpentine channel with obstacles	1 μL min^−1^	15.3 e^−10^	Minimum	Minimum	Maximum
TC20		1 μL min^−1^	15.3 e^−11^	Average	Average	Average
TC21		1 μL min^−1^	15.3 e^−12^	Maximum	Maximum	Minimum
TC22		5 μL min^−1^	15.3 e^−10^	Minimum	Minimum	Maximum
TC23		5 μL min^−1^	15.3 e^−11^	Average	Average	Average
TC24		5 μL min^−1^	15.3 e^−12^	Maximum	Maximum	Minimum
TC25		10 μL min^−1^	15.3 e^−10^	Minimum	Minimum	Maximum
TC26		10 μL min^−1^	15.3 e^−11^	Average	Average	Average
TC27		10 μL min^−1^	15.3 e^−12^	Maximum	Maximum	Minimum

#### 3.2.2 Optimization with fuzzy logic

The selection of input and output variables to be used is the first stage in the fuzzy logic system modelling process. Diffusion control of A and B fluids in the channels is the primary duty of the microfluidic-based micromixer modelled in this work. The output parameters of the diffusion-related fuzzy logic approach must be taken into consideration in order to do this, and the rules must be expressed clearly. Using the fuzzy logic application, optimization procedures are undertaken in this study according to the input and output parameters.

**Fig. 7:**
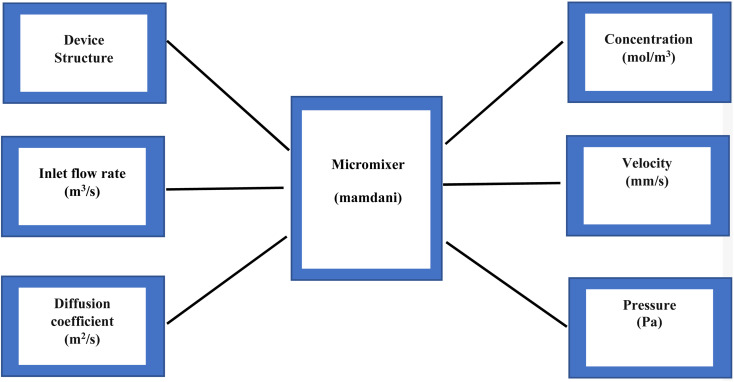
Fuzzy logic model of the micromixer.

The diffusion coefficient and inlet flow rate of the A and B fluids entering the micromixer are the parameters that make up the system input. The system output parameters are the velocity, pressure, and concentration of the liquids. The diffusion of liquids A and B is made possible by the values of the diffusion coefficient. The pressure and velocity of fluids in the micromixer channel are also influenced by the inlet flow rate ratio. Fig. 7 displays the inputs and outputs of the fuzzy logic system.

According to the values of the upper and lower limits of the input and output parameters, the membership function values written for each input and output value are updated in the fuzzy logic approach. The COMSOL Multiphysics application has been used for dozens of different analytical procedures. The results of the analysis are used to develop rules and parameter values. Nine criteria were developed to specify the connection between the parameters after the upper and lower bounds for modelling the necessary parameters using the membership function were chosen. The following table is the fuzzy logic test case (Table 1). We chose three different structures of micromixer devices: Y-shaped straight channel micromixer (SCM), Y-shaped herringbone serpentine shape micromixer (HBM) and Y-shaped herringbone serpentine shape micromixer with obstacles (HBM-OB). Each device structure has two input parameters of flow rate (1, 5 and 10 μL min^−1^), diffusion coefficient (15.3 e^−10^, 15.3 e^−11^ and 15.3 e^−12^ m^2^ s^−1^), and three output parameters of velocity, pressure and concentration. We chose 27 test cases using the fuzzy logic test case table below.

## 4 Results and discussion

### 4.1 Velocity profile of the device

Analyses of the microfluidic based micromixer device were performed using COMSOL Multiphysics software. Twenty-seven different analyses were performed to achieve optimum results for the device. The system input variables are the diffusion coefficient and inlet flow rate, while the outputs are velocity, pressure, and concentration. In this work, to study the mixing performance of the micromixer, water is chosen as the input fluid for both inlets A and B with different concentrations. The properties of the input fluid are as follows: density is 1000 kg m^−3^, viscosity is 0.001 Pa s and the molecular diffusivity (*D*) is 15.3 × 10^−10^ m^2^ s^−1^, 15.3 × 10^−11^ m^2^ s^−1^, 15.3 × 10^−12^ m^2^ s^−1^. The inflow velocity of the fluid in both inlets is considered to be the same (1, 5, 10 μL min^−1^) and the fluid concentrations (*c*) in inlets A and B are taken as 1 mol m^−3^ and 10 mol m^−3^, respectively.

**Fig. 8:**
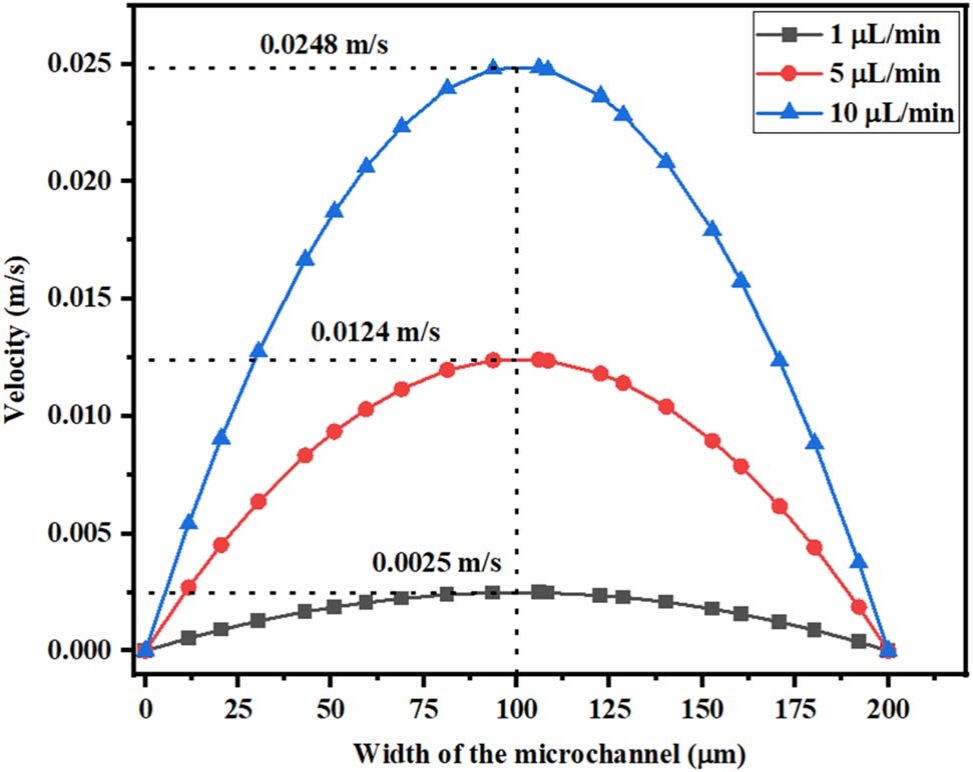
Velocity across the Y-shaped straight channel micromixer.

Fig. 8 shows the velocity across the Y-shaped straight channel micromixer with different flow rates of 1 μL min^−1^, 5 μL min^−1^ and 10 μL min^−1^. The peak velocity was achieved in the middle of the microchannel and the velocity was reduced at the wall of the microfluidic channel due to fluid sticking onto the channel wall.

**Fig. 9:**
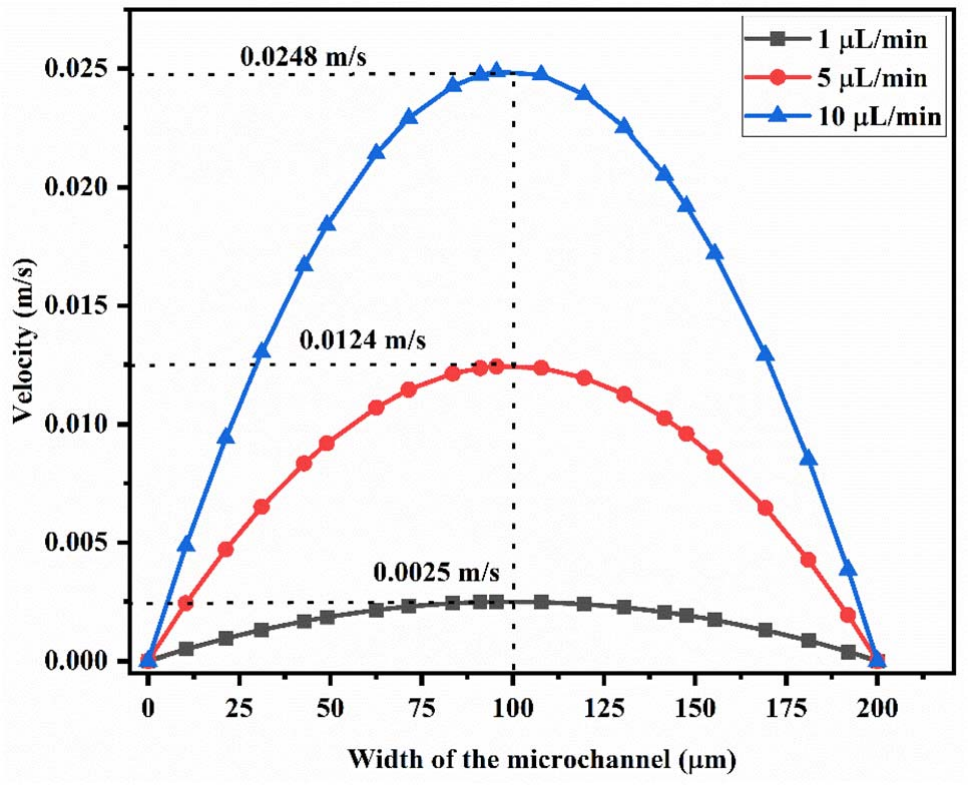
Velocity across the Y-shaped herringbone serpentine channel micromixer.

Fig. 9 shows the velocity across the Y-shaped herringbone serpentine channel micromixer with different flow rates of 1 μL min^−1^, 5 μL min^−1^ and 10 μL min^−1^.

**Fig. 10:**
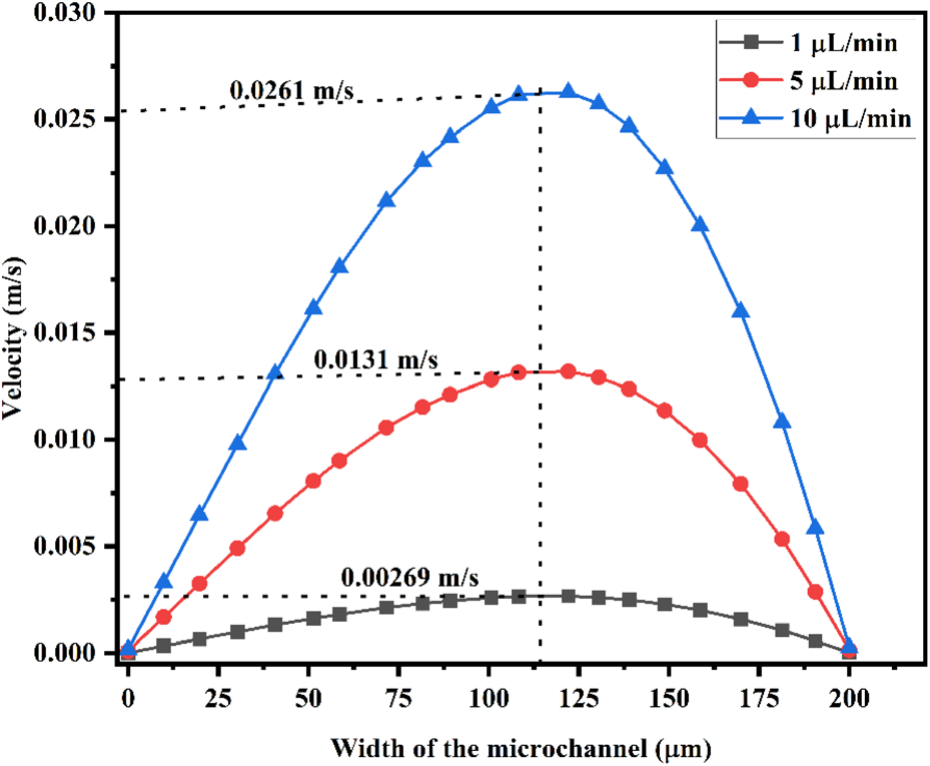
Velocity across the Y-shaped herringbone serpentine channel micromixer with obstacles.

The peak velocity was achieved in the middle of the microchannel and the velocity was reduced at the wall of the microfluidic channel due to fluid sticking onto the channel wall. Fig. 10 shows the velocity across the Y-shaped herringbone serpentine channel micromixer with obstacles, with different flow rates of 1 μL min^−1^, 5 μL min^−1^ and 10 μL min^−1^. The peak velocity was achieved in the middle of the microchannel and the velocity was reduced at the wall of the microfluidic channel due to fluid sticking onto the channel wall.

### 4.2 Concentration analysis across the device

**Fig. 11:**
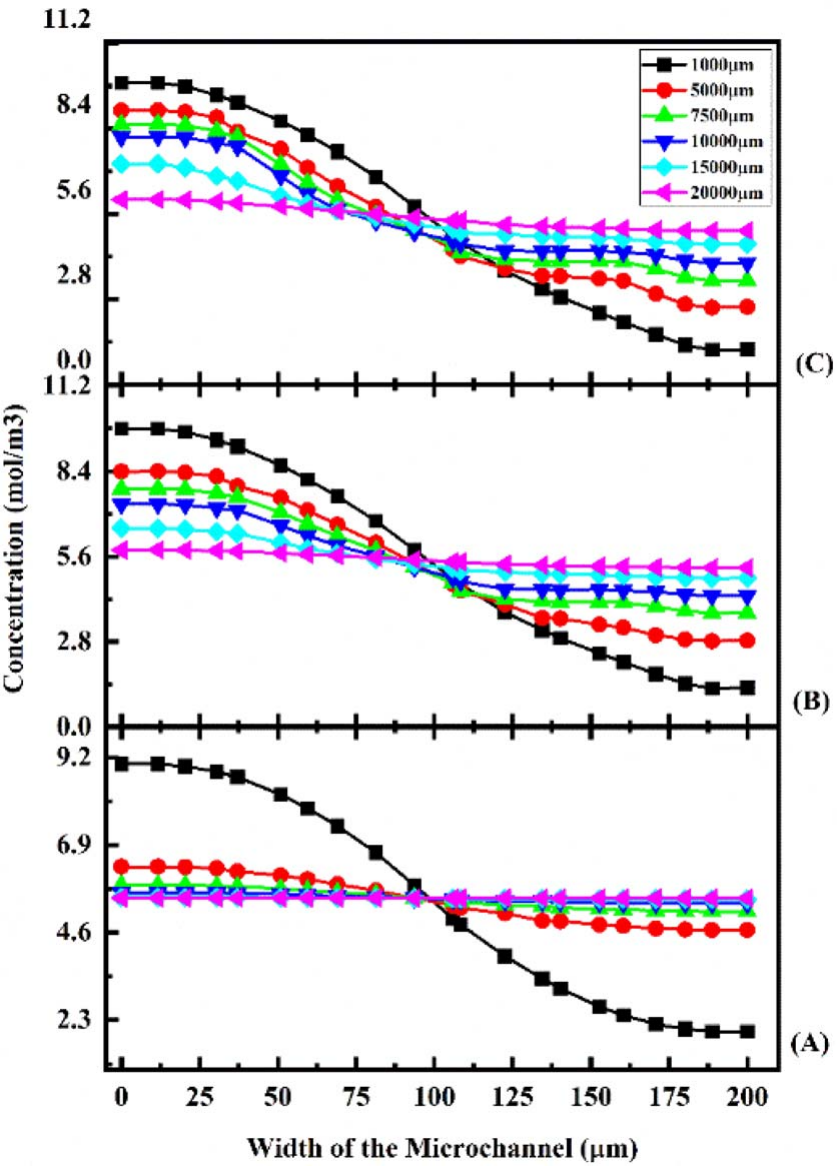
Concentration across the Y-shaped straight channel micromixer at 1 μL min^−1^ (A), 5 μL min^−1^ (B), and 10 μL min^−1^ (C) with a diffusion co-efficient of 15.3 × 10^−10^ m^2^ s^−1^.

A concentration study of the device was carried out with different test cases as given in Table 1. Fig. 11 shows the concentration across the Y-shaped straight channel micromixer at different flow rates of 1 μL min^−1^ (11(A)), 5 μL min^−1^ (11(B)), and 10 μL min^−1^ (11(C)) with a diffusion co-efficient of 15.3 × 10^−10^ m^2^ s^−1^.

Fig. 11(A), (B) and (C) show the concentration across the fluidic channel at different locations of 1000 μm, 5000 μm, 7500 μm, 10 000 μm, 15 000 μm and 20 000 μm. When fluids enter into the straight channel from the inlets, the fluid flow is laminar and the fluid–fluid interaction time is greater when the fluid flow is at a low flow rate of 1 μL min^−1^ (11(A)), so better mixing concentration is observed. Similarly, when the fluid flow is increased to 5 μL min^−1^ (11(B)) and 10 μL min^−1^ (11(C)), the fluid–fluid interaction is reduced, so the mixing concentration level is reduced at different locations.

**Fig. 12:**
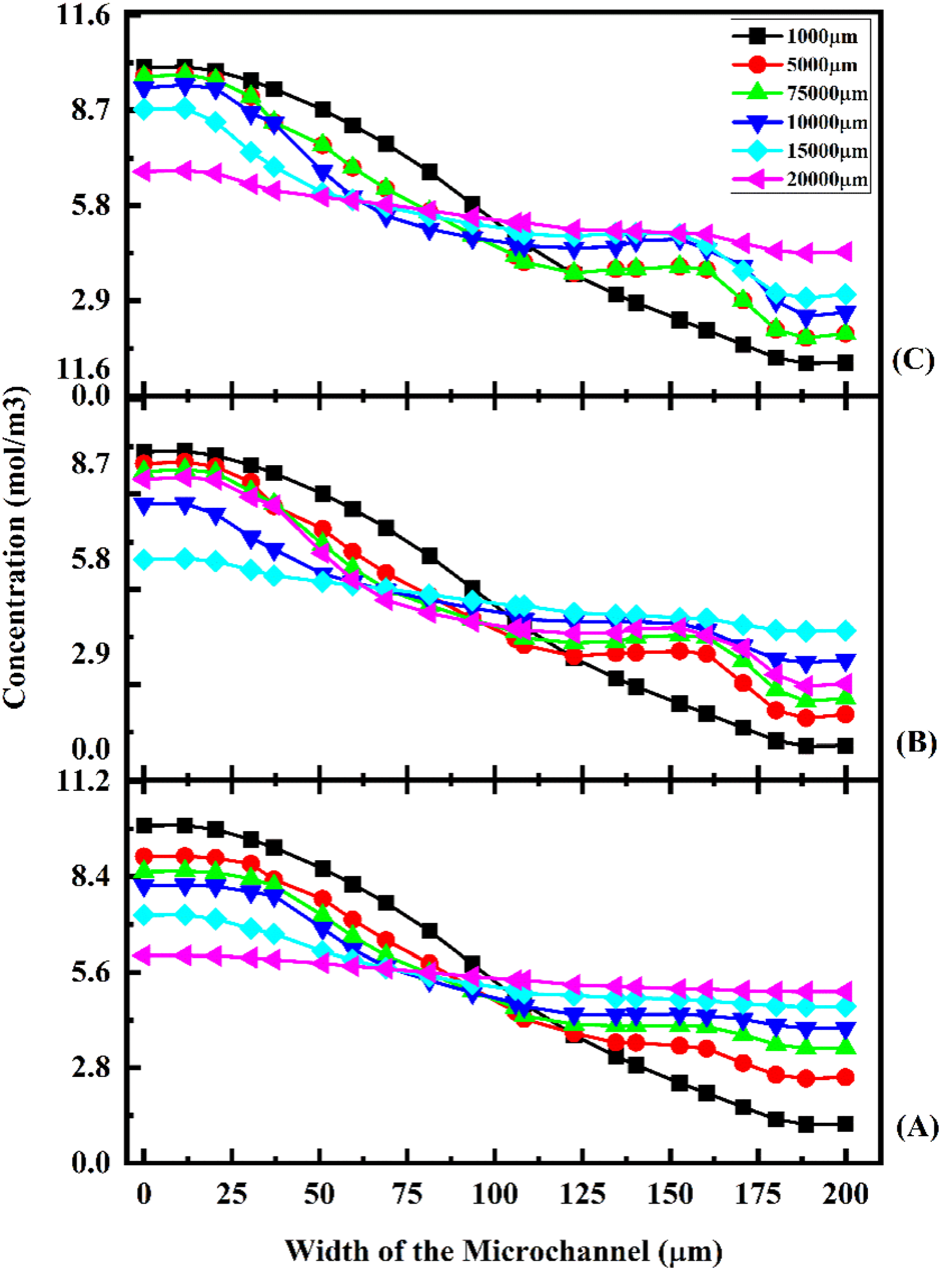
Concentration across the Y-shaped straight channel micromixer at 1 μL min^−1^ (A), 5 μL min^−1^ (B), and 10 μL min^−1^ (C) with a diffusion co-efficient of 15.3 × 10^−11^ m^2^ s^−1^.

Fig. 12 shows the concentration across the Y-shaped straight channel micromixer at different flow rates of 1 μL min^−1^ (12(A)), 5 μL min^−1^ (12(B)), and 10 μL min^−1^ (12(C)) with a diffusion co-efficient of 15.3 × 10^−11^ m^2^ s^−1^. At different locations along the fluidic channel (1000 μm, 5000 μm, 7500 μm, 10 000 μm, 15 000 μm, and 20 000 μm), Fig. 12(A), (B) and (C) show the concentrations along the fluidic channel at different locations. In a straight channel, fluid enters from the inlets in a parallel flow. The observed mixing concentration is higher when the fluid flow rate is 1 μL min^−1^ (12(A)), which results from greater fluid–fluid interaction time.

Similarly, the mixing concentration level is reduced at different locations when the fluid flow is increased to 5 μL min^−1^ (12(B)) and 10 μL min^−1^ (12(C)). The mixing concentrations of test cases TC4, TC5 and TC6 are comparatively lower than the previous test cases TC1, TC2 and TC3.

**Fig. 13:**
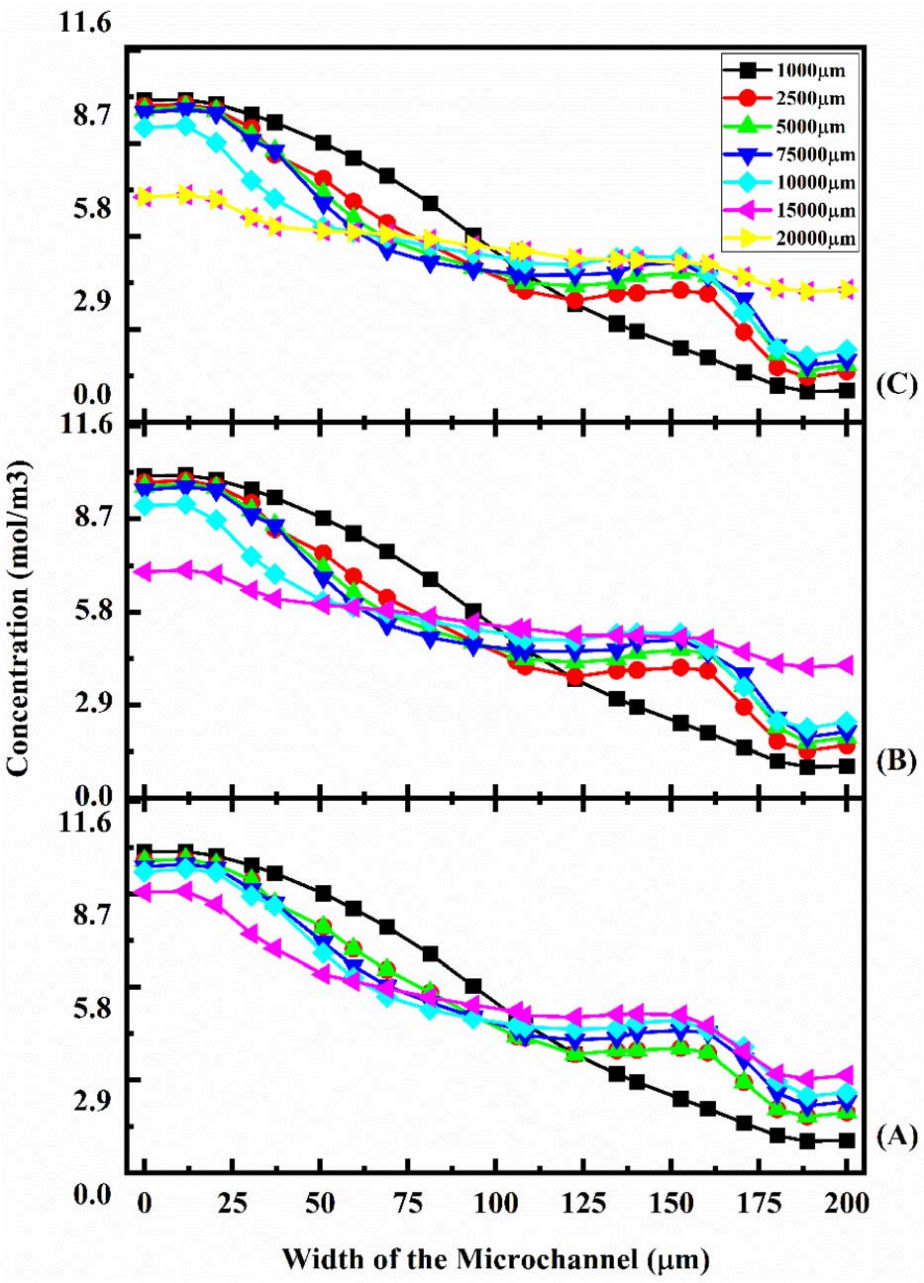
Concentration across the Y-shaped straight channel micromixer at 1 μL min^−1^ (A), 5 μL min^−1^ (B), and 10 μL min^−1^ (C) with a diffusion co-efficient of 15.3 × 10^−12^ m^2^ s^−1^.

Fig. 13 shows the concentration across the Y-shaped straight channel micromixer at different flow rates of 1 μL min^−1^ (13(A)), 5 μL min^−1^ (13(B)), and 10 μL min^−1^ (13(C)) with a diffusion co-efficient of 15.3 × 10^−12^ m^2^ s^−1^. In Fig. 13(A), (B) and (C), the concentration across the fluidic channel is depicted at various points of 1000 μm, 5000 μm, 7500 μm, 10 000 μm, 15 000 μm, and 20 000 μm.

**Fig. 14:**
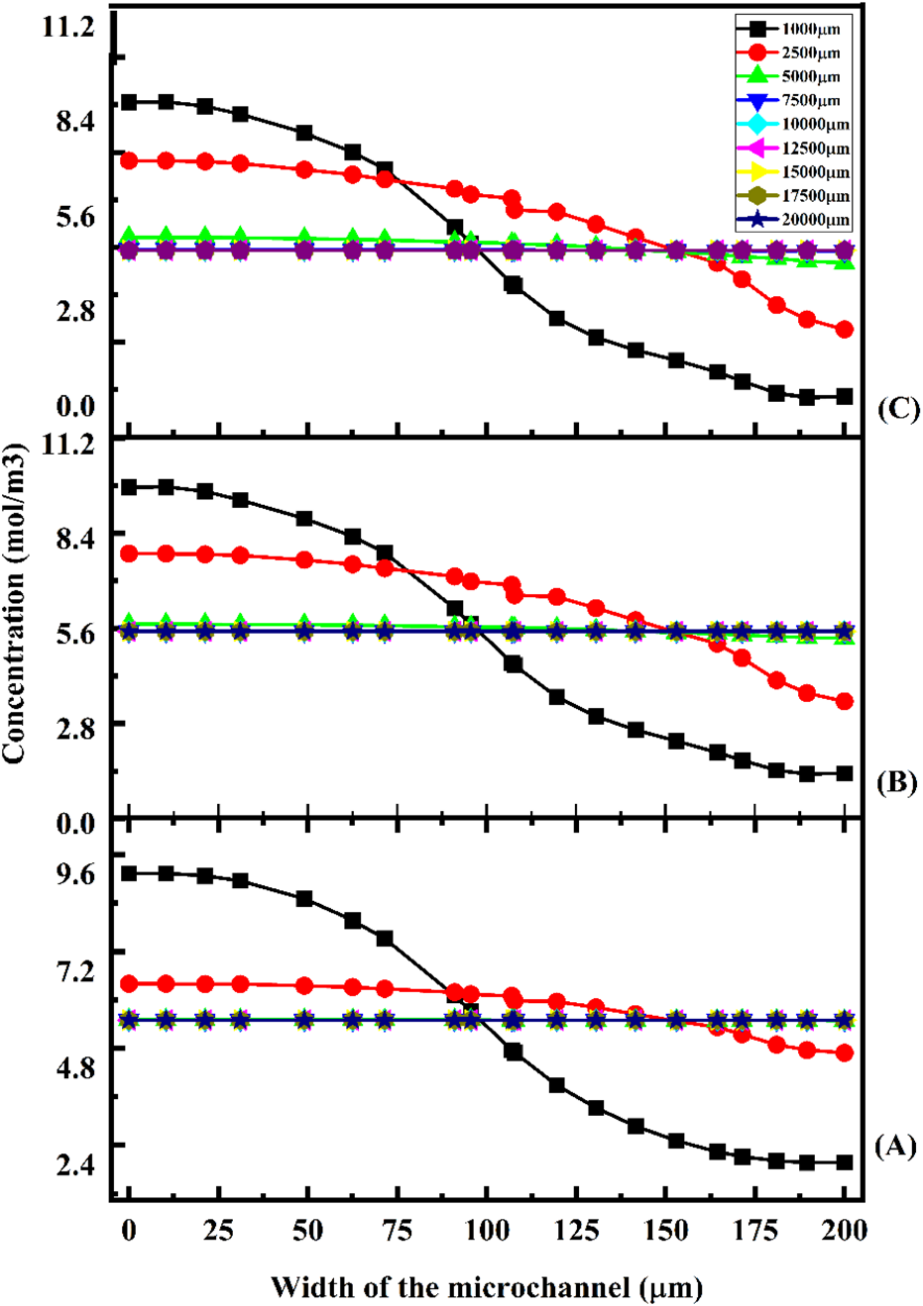
Concentration across the Y-shaped herringbone serpentine channel micromixer at 1 μL min^−1^ (A), 5 μL min^−1^ (B), and 10 μL min^−1^ (C) with a diffusion co-efficient of 15.3 × 10^−10^ m^2^ s^−1^.

**Fig. 15:**
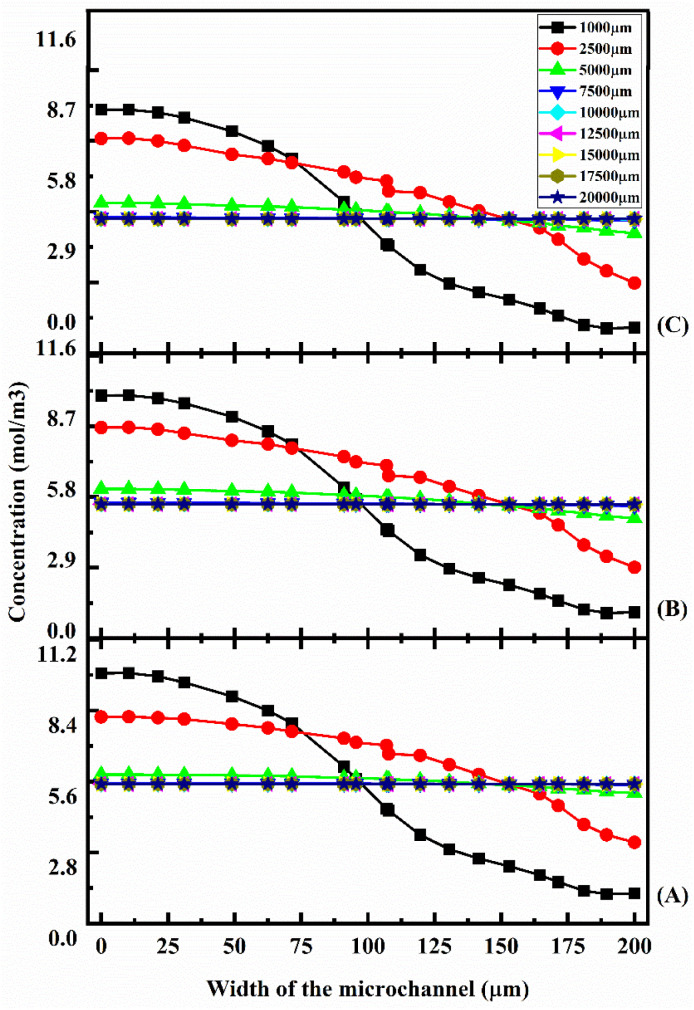
Concentration across the Y-shaped herringbone serpentine channel micromixer at 1 μL min^−1^ (A), 5 μL min^−1^ (B), and 10 μL min^−1^ (C) with a diffusion co-efficient of 15.3 × 10^−11^ m^2^ s^−1^.

Fluid flow is laminar when it enters the straight channel from the inlets. At low flow rates, such as 1 μL min^−1^ (13(A)), the fluid–fluid interaction time is greater, resulting in a better mixing concentration. Additionally, as the fluid flow increases to 5 μL min^−1^ (13(B)) and 10 μL min^−1^ (13(C)), the fluid–fluid interaction reduces, thereby decreasing the mixing concentration at different locations. Fig. 14 shows the concentration across the Y-shaped herringbone serpentine channel micromixer at different flow rates of 1 μL min^−1^ (14(A)), 5 μL min^−1^ (14(B)), and 10 μL min^−1^ (14(C)) with a diffusion co-efficient of 15.3 × 10^−10^ m^2^ s^−1^. This figure illustrates the concentration of fluids across the fluidic channel across a number of locations of 1000 μm, 5000 μm, 7500 μm, 10 000 μm, 15 000 μm, and 20 000 μm. The fluid flow in a herringbone serpentine channel is laminar when it enters from the inlets. When fluid flow is maintained at a low flow rate of 1 μL min^−1^ (14(A)), the amount of fluid–fluid interaction is greater, resulting in a better mixing concentration. Furthermore, the mixing concentration level is reduced at different locations as the fluid flow increases to 5 μL min^−1^ (14(B)) and 10 μL min^−1^ (14(C)). Fig. 15 shows the concentration across the Y-shaped herringbone serpentine channel micromixer at different flow rates of 1 μL min^−1^ (15(A)), 5 μL min^−1^ (15(B)), and 10 μL min^−1^ (15(C)) with a diffusion co-efficient of 15.3 × 10^−11^ m^2^ s^−1^. At different locations within the fluidic channel, of 1000 μm, 5000 μm, 7500 μm, 10 000 μm, 15 000 μm and 20 000 μm, Fig. 15(A), (B) and (C) illustrate the concentrations.

Laminar flow occurs when fluid is introduced into the herringbone serpentine channel from the inlets. At a low flow rate of 1 μL min^−1^ (15(A)), there is more fluid–fluid interaction time, resulting in a higher mixing concentration. In a similar manner, as the fluid flow increases to 5 μL min^−1^ (15(B)) and 10 μL min^−1^ (15(C)), the fluid–fluid interaction is reduced, which results in a reduction in mixing concentration levels at various locations.

**Fig. 16:**
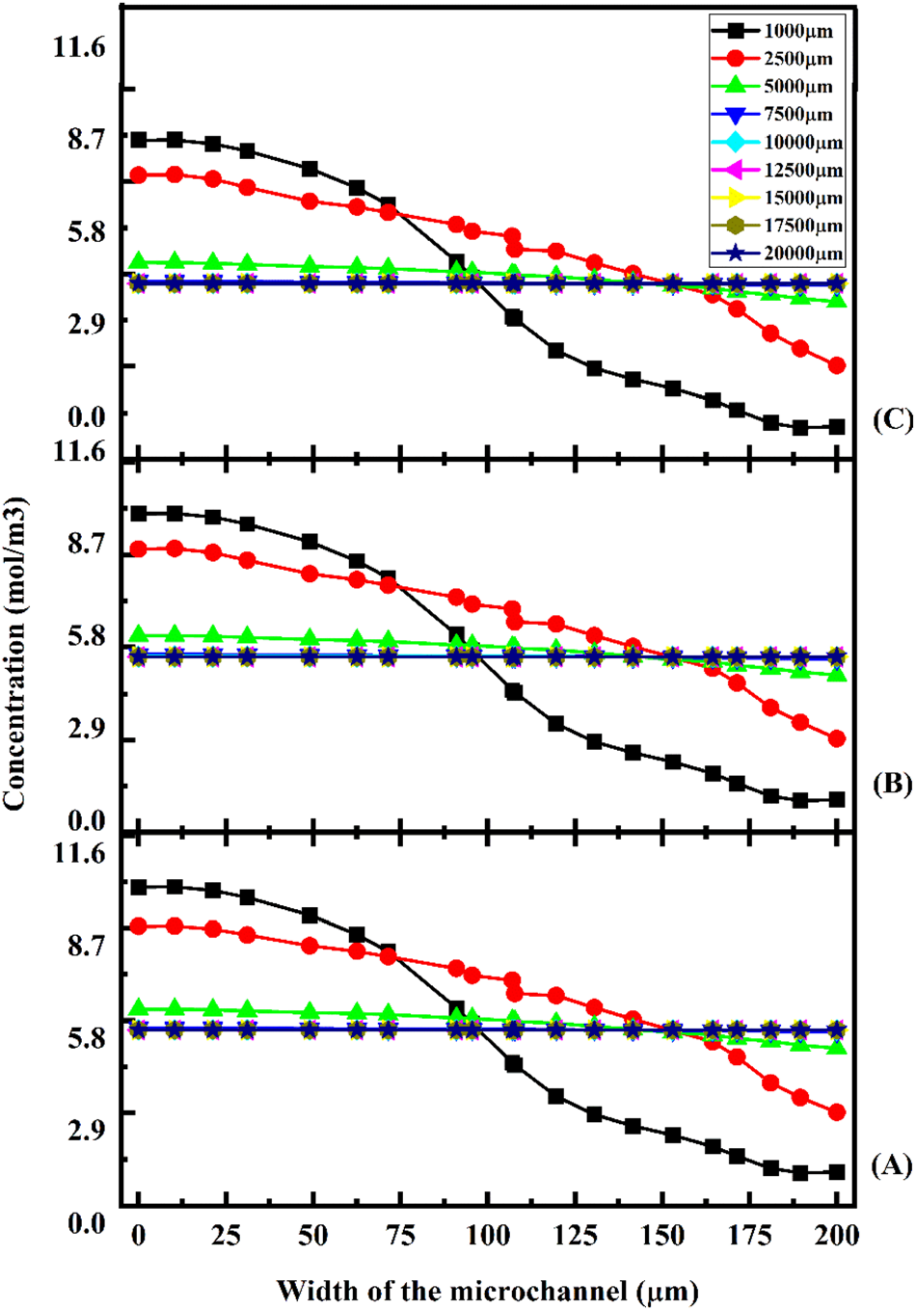
Concentration across the Y-shaped herringbone serpentine channel micromixer at 1 μL min^−1^ (A), 5 μL min^−1^ (B), and 10 μL min^−1^ (C) with a diffusion co-efficient of 15.3 × 10^−12^ m^2^ s^−1^.

Fig. 16 shows the concentration across the Y-shaped herringbone serpentine channel micromixer at different flow rates of 1 μL min^−1^ (16(A)), 5 μL min^−1^ (16(B)), and 10 μL min^−1^ (16(C)) with a diffusion co-efficient of 15.3 × 10^−12^ m^2^ s^−1^. In Fig. 16, the concentrations are depicted at different points in the fluidic channel for different distances of 1000 μm, 5000 μm, 7500 μm, 10 000 μm, 15 000 μm, and 20 000 μm. From the inlets, fluid flows in a laminar fashion through the herringbone serpentine channel. With a low fluid flow rate of 1 μL min^−1^ (16(A)), the fluid–fluid interaction time is greater, resulting in a better mixing concentration. In the same way, as the fluid flow increases to 5 μL min^−1^ (16(B)) and 10 μL min^−1^ (16(C)), the fluid–fluid interaction is reduced, resulting in a reduction in mixing concentration levels at differing locations.

**Fig. 17:**
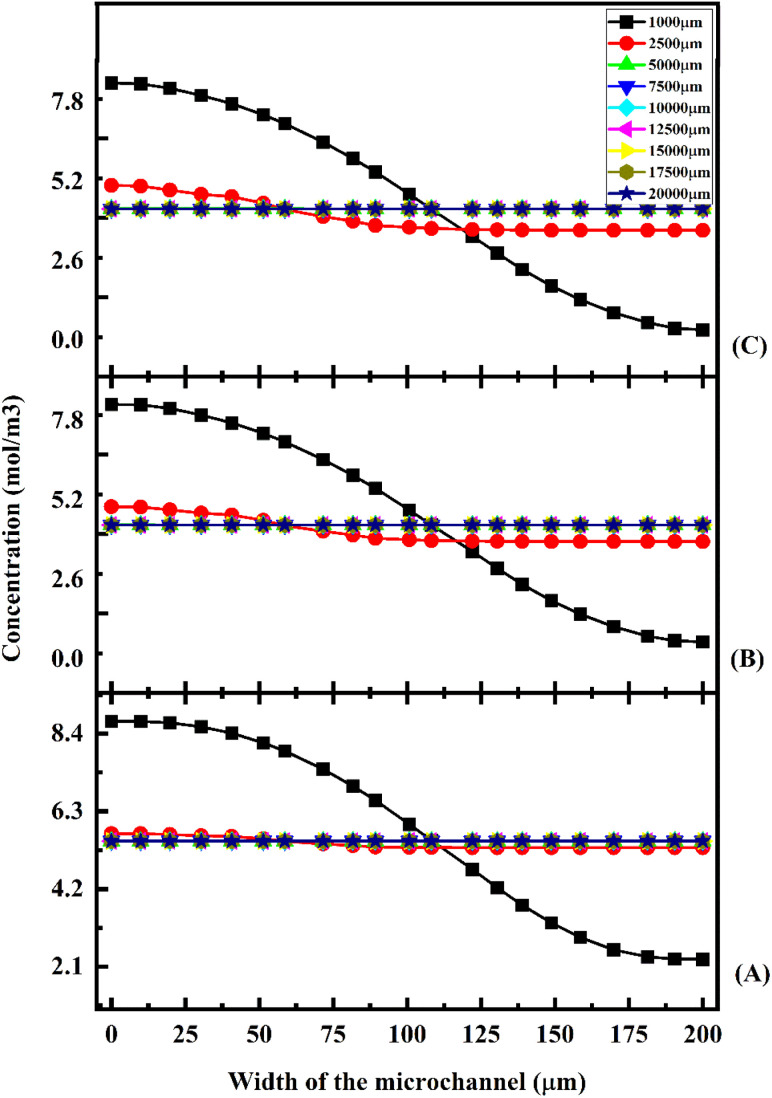
Concentration across the Y-shaped herringbone serpentine channel micromixer with obstacles at 1 μL min^−1^ (A), 5 μL min^−1^ (B), and 10 μL min^−1^ (C) with a diffusion co-efficient of 15.3 × 10^−10^ m^2^ s^−1^.

Fig. 17 shows the concentration across the fluidic channel at different locations of 1000 μm, 5000 μm, 7500 μm, 10 000 μm, 15 000 μm and 20 000 μm with different flow rates of 1 μL min^−1^ (17(A)), 5 μL min^−1^ (17(B)), and 10 μL min^−1^ (17(C)) with a diffusion co-efficient of 15.3 × 10^−10^ m^2^ s^−1^. In the present study, we observe that a laminar fluid flow is observed when fluid enters the micromixer devices from the inlets. A higher mixing concentration is observed when the flow rate is low at 1 μL min^−1^ (17(A)), so the fluid–fluid interaction time is greater. Furthermore, an increase in fluid flow rate decreases the time required for fluid–fluid interaction, resulting in a decrease in mixing concentration. For the proposed device, quadrant-shaped obstacles are introduced inside the microchannel for improved mixing within a short period of time, which allows for a reduction in the length of the device. It is considered that the mixing process has been completed once the fluid concentration reaches the average concentration of the fluid inflow (5.5 mol m^−3^). According to the results, at a flow rate of 1 μL min^−1^, the concentration level is saturated throughout a distance of 5000 μm.

This study shows that a better mixing concentration is achieved compared with the other two micromixer devices: Y-shaped straight channel micromixer and Y-shaped herringbone serpentine shape micromixer without obstacles. When the flow rate is increased to 5 and 10 μL min^−1^ (17(B) and 17(C)), the mixing concentration level is reduced slightly compared with the other two micromixer devices.

**Fig. 18:**
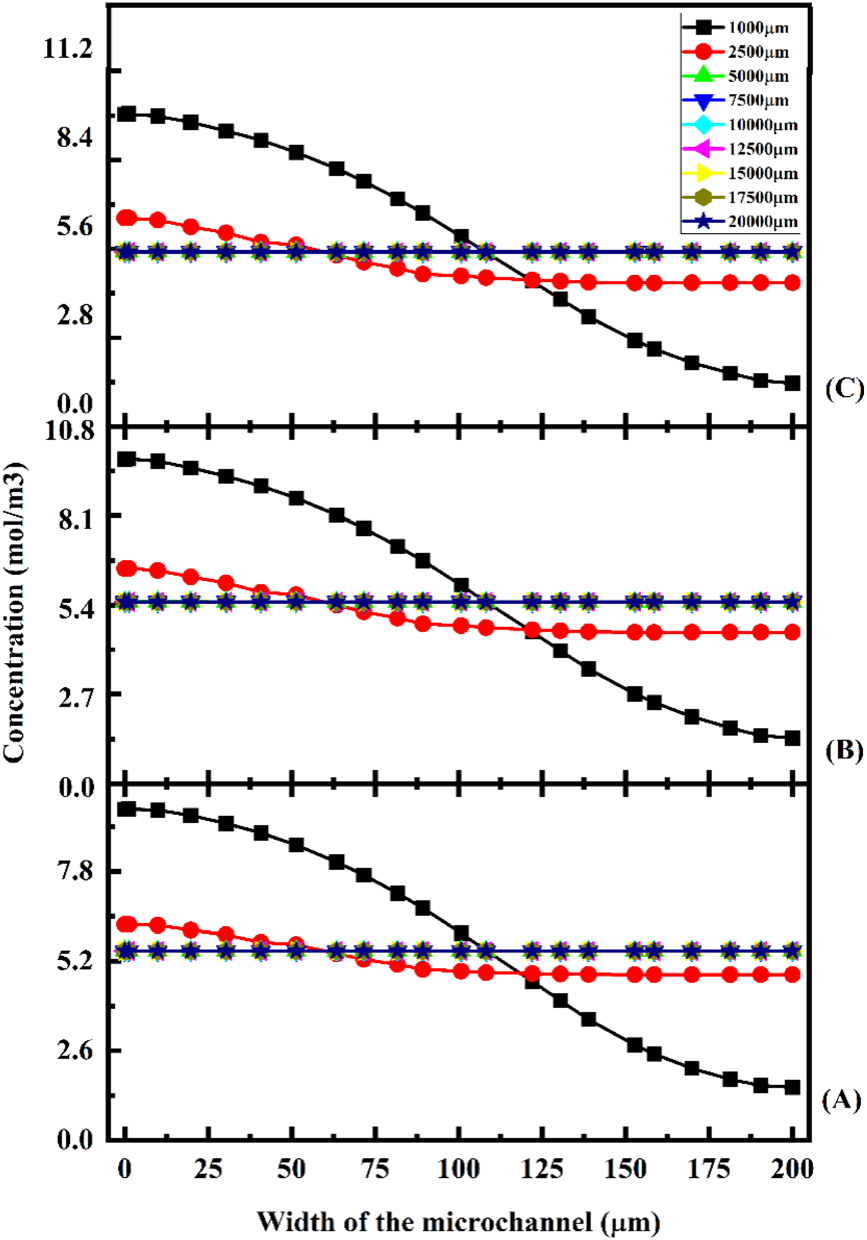
Concentration across the Y-shaped herringbone serpentine channel micromixer with obstacles at 1 μL min^−1^ (A), 5 μL min^−1^ (B), and 10 μL min^−1^ (C) with a diffusion co-efficient of 15.3 × 10^−11^ m^2^ s^−1^.

Fig. 18 shows the concentration across the fluidic channel at different locations of 1000 μm, 5000 μm, 7500 μm, 10 000 μm, 15 000 μm and 20 000 μm with different flow rates of 1 μL min^−1^ (18(A)), 5 μL min^−1^ (18(B)), and 10 μL min^−1^ (18(C)) with a diffusion co-efficient of 15.3 × 10^−11^ m^2^ s^−1^. In this study, we can observe that when fluids enter into micromixer device from the inlets, the fluid flow is laminar and fluid–fluid interaction time is greater when the fluid flow is at low flow rate of 1 μL min^−1^ (18(A)) so a better mixing concentration is observed. Similarly, when the fluid flow rate is increased, the fluid–fluid interaction time is reduced because the mixing concentration is reduced. The proposed device has quadrant shaped obstacles introduced inside the microchannel for better mixing within a short duration, reducing the length of the device. It is discovered that the concentration level is saturated throughout a 5000 μm length at a flow rate of 1 μL min^−1^.

**Fig. 19:**
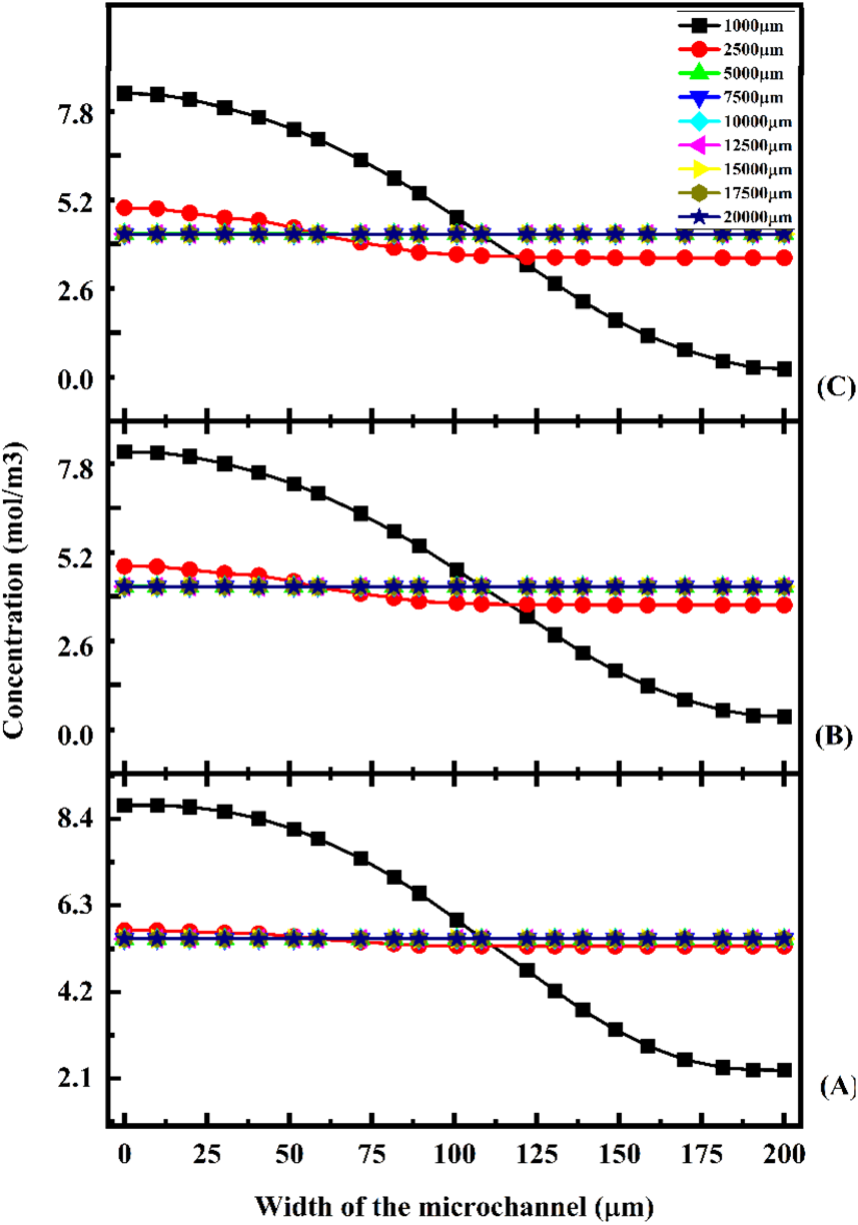
Concentration across the Y-shaped herringbone serpentine channel micromixer with obstacles at 1 μL min^−1^ (A), 5 μL min^−1^ (B), and 10 μL min^−1^ (C) with a diffusion co-efficient of 15.3 × 10^−12^ m^2^ s^−1^.

This study shows that better mixing concentration is achieved compared with the other two micromixer devices: Y-shaped straight channel micromixer and Y-shaped herringbone serpentine shape micromixer without obstacles. When the flow rate is increased to 5 and 10 μL min^−1^ (18(B) and 18(C)), the mixing concentration level is reduced slightly compared with the other two micromixer devices. Fig. 19 shows the concentration across the fluidic channel at different locations of 1000 μm, 5000 μm, 7500 μm, 10 000 μm, 15 000 μm and 20 000 μm with different flow rates of 1 μL min^−1^ (19(A)), 5 μL min^−1^ (19(B)), and 10 μL min^−1^ (19(C)) with a diffusion co-efficient of 15.3 × 10^−12^ m^2^ s^−1^. It was observed that the fluid flow into the micromixer device is laminar when it enters the mixing zone. When the fluid flow is low, 1 μL min^−1^ (19(A)), the fluid–fluid interaction time is greater, so the mixing concentration is better. When the flow rate is low (19(A)), the fluid–fluid interaction time is longer, resulting in a better mixing concentration. Similarly, an increasing flow rate reduces the fluid–fluid interaction time, which reduces the mixing concentration. An obstacle of quadrant shape has been introduced into the microchannel for better mixing within a short period of time and to reduce the size of the device. The concentration level is saturated over a length of 5000 μm by mixing at a rate of 1 μL min^−1^. In this study, it was shown that the mixing concentration was higher compared with the other two micromixers: Y-shaped straight channel micromixers and Y-shaped herringbone serpentine micromixers without obstructions. The mixing concentration level is slightly reduced when the flow rate increases to 5 μL min^−1^ and 10 μL min^−1^ (19(B) and 19(C)), in comparison with the other two micromixers.

### 4.3 Mixing efficiency of the devices

**Fig. 20:**
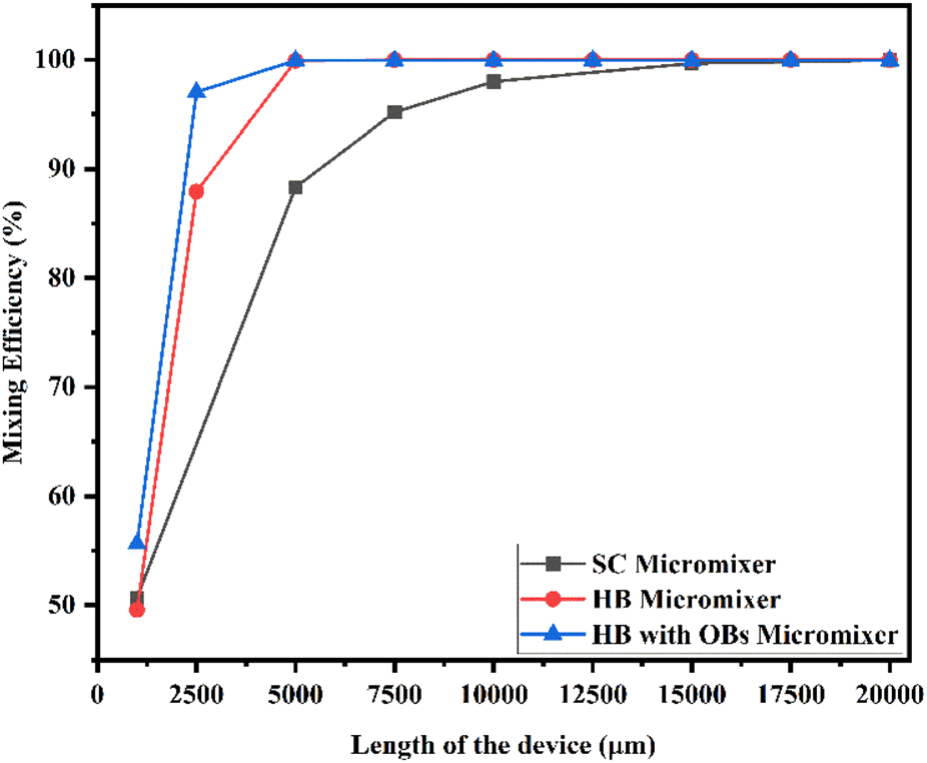
Mixing efficiency across the micromixer devices at 1 μL min^−1^ with a diffusion co-efficient of 15.3 × 10^−10^ m^2^ s^−1^.

Fig. 20 shows the mixing efficiency across the fluidic channel at different locations of 1000 μm, 2500 μm, 5000 μm, 7500 μm, 10 000 μm, 12 500 μm, 15 000 μm, 17 500 μm and 20 000 μm with a constant flow rate and a diffusion co-efficient of 15.3 × 10^−10^ m^2^ s^−1^.

From Fig. 20, we are able to see three different micromixer devices: Y-shaped straight channel micromixer (SCM), Y-shaped herringbone serpentine shape micromixer (HBM) and Y-shaped herringbone serpentine shape micromixer with obstacles (HBM-OB).

In this study, we can observe that the mixing efficiencies of the SCM device at the above-mentioned locations are 50.68%, 88.33%, 95.17%, 97.98%, 99.66% and 99.96%. The mixing efficiencies of the HBM device are 49.55%, 87.93%, 99.89%, 99.97%, 99.97%, 99.97%, 99.97%, 99.97% and 99.97%. Similarly, the mixing efficiencies of HBM-OB are 55.62%, 97.02%, 99.93%, 99.93%, 99.93%, 99.93%, 99.93%, 99.93% and 99.93%. When comparing the mixing efficiency of all three types of micromixer device and mixing length, the best mixing efficiency was achieved in the HBM-OB device due to the structural dimensions of the device and the obstacles. The obstacle-induced fluid–fluid interaction caused better mixing, which was achieved in a short duration compared with the other two types of micromixer device.

**Fig. 21:**
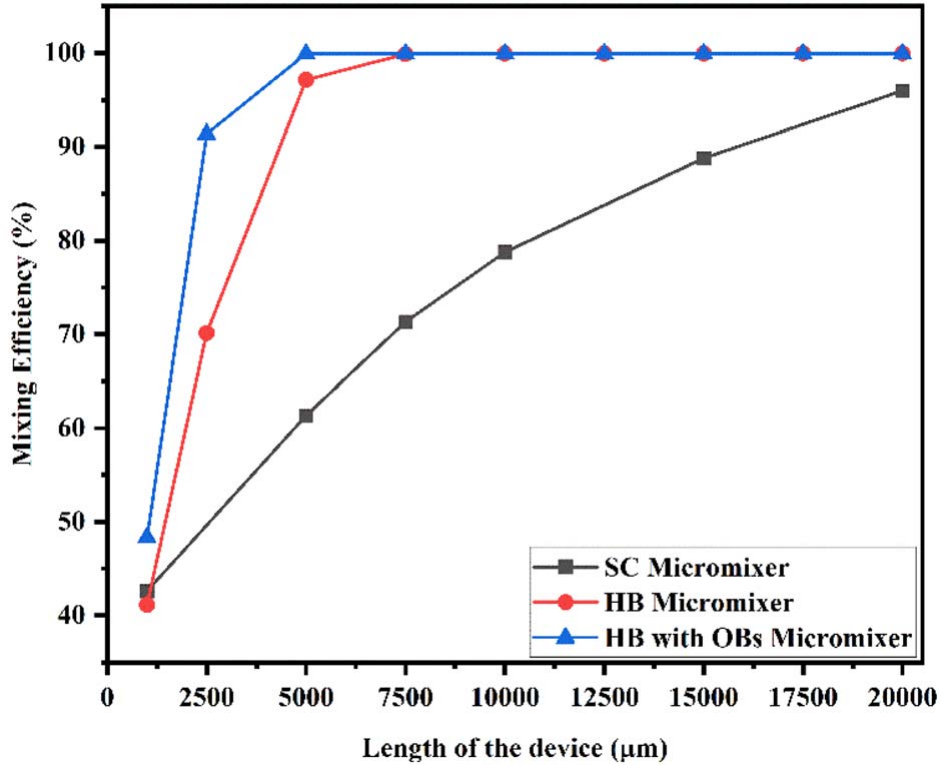
Mixing efficiency across the micromixer devices at 1 μL min^−1^ with a diffusion co-efficient of 15.3 × 10^−11^ m^2^ s^−1^.

Fig. 21 shows the mixing efficiency across the fluidic channel at different locations of 1000 μm, 2500 μm, 5000 μm, 7500 μm, 10 000 μm, 12 500 μm, 15 000 μm, 17 500 μm and 20 000 μm with a constant flow rate and a diffusion co-efficient of 15.3 × 10^−11^ m^2^ s^−1^. In Fig. 21, we can see three different Y-shaped micromixer devices: a Y-shaped straight channel micromixer (SCM), a Y-shaped herringbone serpentine shape micromixer (HBM), and a Y-shaped herringbone serpentine shape micromixer with obstacles (HBM-OB). During this study, we could observe that the mixing efficiencies of the SCM device at the above-mentioned locations were 42.58%, 61.33%, 71.30%, 78.74%, 88.78% and 95.96%, respectively. In terms of mixing efficiency, 41.12%, 70.12%, 97.14%, 99.89%, 99.95%, 99.95%, 99.95%, 99.95%, 99.95% and 99.95% were achieved for the HBM device. As for HBM-OB, the mixing efficiencies were 48.33%, 91.37%, 99.92%, 99.93%, 99.93%, 99.93%, 99.93%, 99.93% and 99.93%. A comparison of the mixing efficiency of all three types of micromixer device and mixing length revealed that the HBM-OB device had the best mixing efficiency, owing to the structure and the obstacles of the device. In comparison with the other two types of micromixer device, the obstacles induce fluid–fluid interaction, which results in improved mixing in a short period of time.

**Fig. 22:**
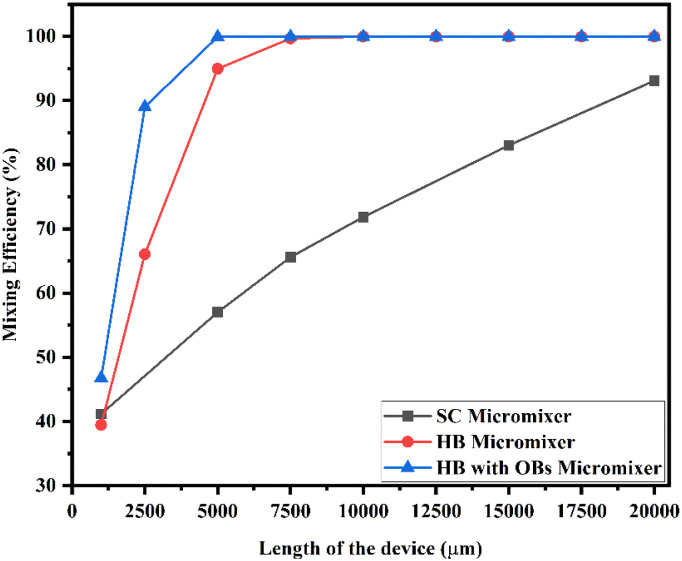
Mixing efficiency across the micromixer devices at 1 μL min^−1^ with a diffusion co-efficient of 15.3 × 10^−12^ m^2^ s^−1^.

Fig. 22 shows the mixing efficiency across the fluidic channel at different locations of 1000 μm, 2500 μm, 5000 μm, 7500 μm, 10 000 μm, 12 500 μm, 15 000 μm, 17 500 μm and 20 000 μm with a constant flow rate and a diffusion co-efficient of 15.3 × 10^−12^ m^2^ s^−1^. From Fig. 22, we are able to see three different micromixer devices: Y-shaped straight channel micromixer (SCM), Y-shaped herringbone serpentine shape micromixer (HBM) and Y-shaped herringbone serpentine shape micromixer with obstacles (HBM-OB). In this study, we can observe that the mixing efficiencies of the SCM device at the above-mentioned locations are 41.15%, 57.02%, 65.59%, 71.84%, 83.01% and 93.07%. The mixing efficiencies of the HBM device are 39.46%, 66.06%, 94.94%, 99.68%, 99.94%, 99.95%, 99.95%, 99.95%, and 99.95%. Similarly, the mixing efficiencies of HBM-OB are 46.77%, 89.01%, 99.91%, 99.94%, 99.94%, 99.94%, 99.94%, 99.94% and 99.94%. When comparing the mixing efficiency of all three types of micromixer device and mixing length, the best mixing efficiency was achieved in the HBM-OB device due to the structural dimensions of the device and the obstacles. The obstacles induce fluid–fluid interaction, which causes better mixing, which was achieved over a short duration compared with the other two types of micromixer device.

**Fig. 23:**
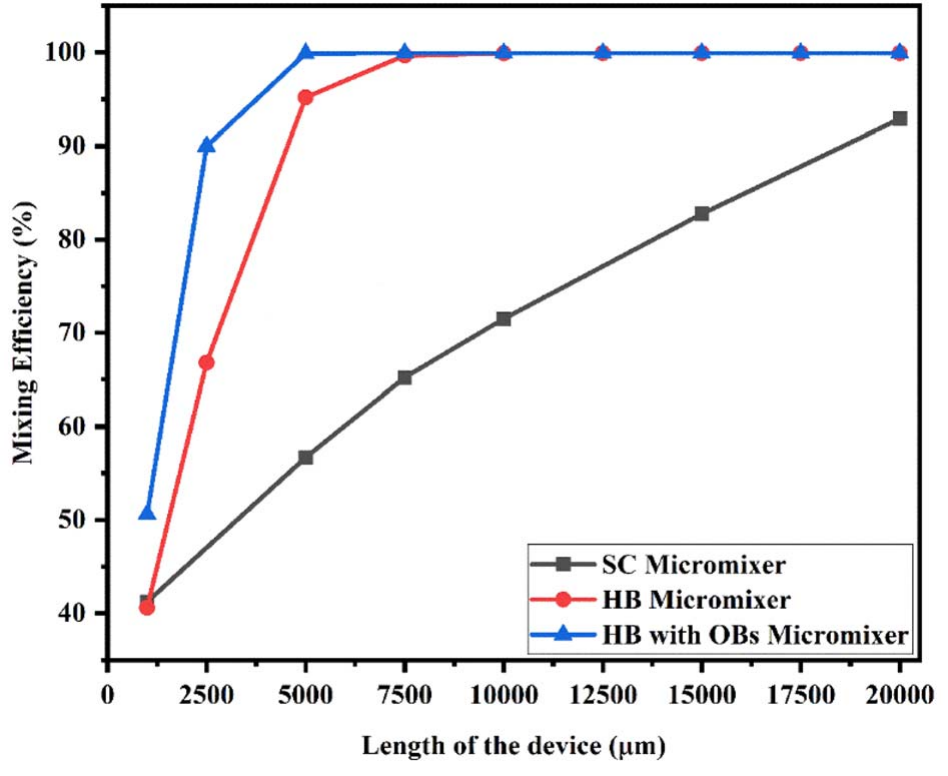
Mixing efficiency across the micromixer devices at 5 μL min^−1^ with a diffusion co-efficient of 15.3 × 10^−10^ m^2^ s^−1^.

Fig. 23 shows the mixing efficiency across the fluidic channel at different locations of 1000 μm, 2500 μm, 5000 μm, 7500 μm, 10 000 μm, 12 500 μm, 15 000 μm, 17 500 μm and 20 000 μm with a constant flow rate and a diffusion co-efficient of 15.3 × 10^−10^ m^2^ s^−1^. From Fig. 23, we are able to see three different micromixer devices: Y-shaped straight channel micromixer (SCM), Y-shaped herringbone serpentine shape micromixer (HBM) and Y-shaped herringbone serpentine shape micromixer with obstacles (HBM-OB).

In this study, we can observe that the mixing efficiencies of the SCM device at the above-mentioned locations are 41.21%, 56.69%, 65.23%, 71.50%, 82.72% and 92.88%. The mixing efficiencies of the HBM device are 40.58%, 66.80%, 95.18%, 99.67%, 99.91%, 99.92%, 99.92%, 99.92% and 99.92%. Similarly, the mixing efficiencies of HBM-OB are 50.60%, 89.97%, 99.88%, 99.90%, 99.90%, 99.90%, 99.90%, 99.90% and 99.90%. When the mixing efficiency of all three types of micromixer device and mixing length are compared, the best mixing efficiency was achieved in HBM-OB.

**Fig. 24:**
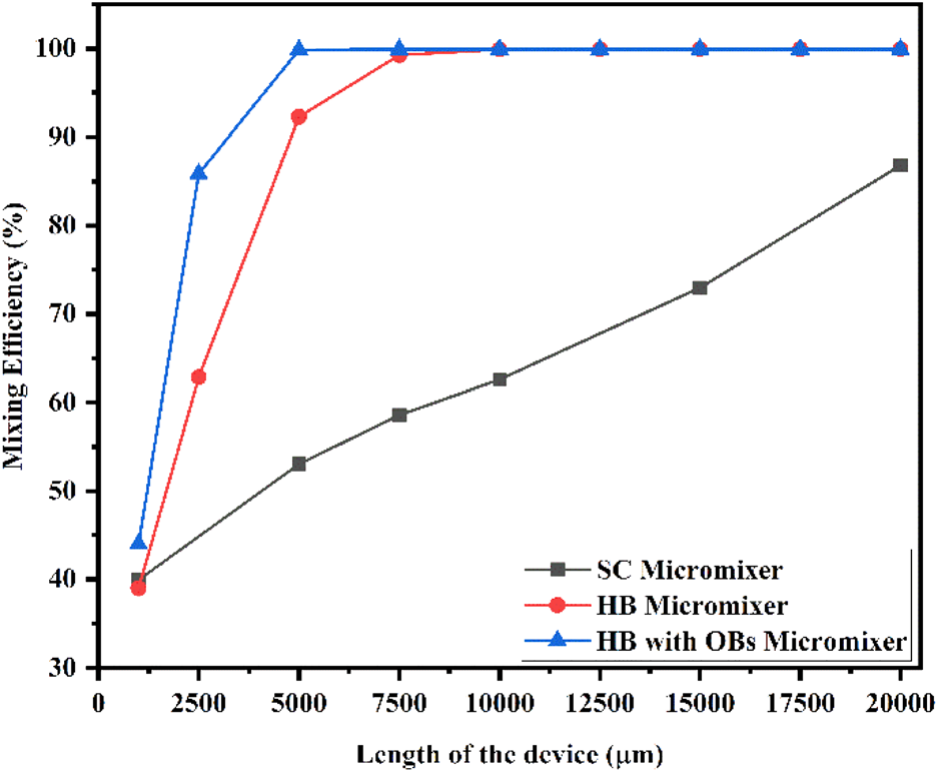
Mixing efficiency across the micromixer devices at 5 μL min^−1^ with a diffusion co-efficient of 15.3 × 10^−11^ m^2^ s^−1^.

Fig. 24 shows the mixing efficiency across the fluidic channel at different locations of 1000 μm, 2500 μm, 5000 μm, 7500 μm, 10 000 μm, 12 500 μm, 15 000 μm, 17 500 μm and 20 000 μm with a constant flow rate and a diffusion co-efficient of 15.3 × 10^−11^ m^2^ s^−1^. This figure displays three different types of micromixers: a Y-shaped straight channel micromixer (SCM), a Y-shaped herringbone serpentine shape micromixer (HBM) and a Y-shaped herringbone serpentine shape micromixer with obstacles (HBM-OB). As a result of this study, we observed 39.94%, 53.05%, 58.60%, 62.59%, 72.95% and 86.81% mixing efficiencies for the SCM device at each of the above-mentioned locations. This graph shows the mixing efficiencies of the HBM device as 38.98%, 62.83%, 92.28%, 99.24%, 99.88%, 99.93%, 99.93%, 99.93% and 99.93%. It was found that the mixing efficiencies of HBM-OB were 43.99%, 85.86%, 99.84%, 99.91%, 99.91%, 99.91%, 99.91%, 99.91%, 99.91%, 99.91% and 99.91%. A comparison of the mixing efficiency of all three types of micromixers and mixing length shows that the HBM-OB device has the best mixing efficiency due to its structural dimensions and the obstacles. Compared with the other two types of micromixer device, the obstacles induced fluid–fluid interaction, resulting in better mixing in a short period of time.

**Fig. 25:**
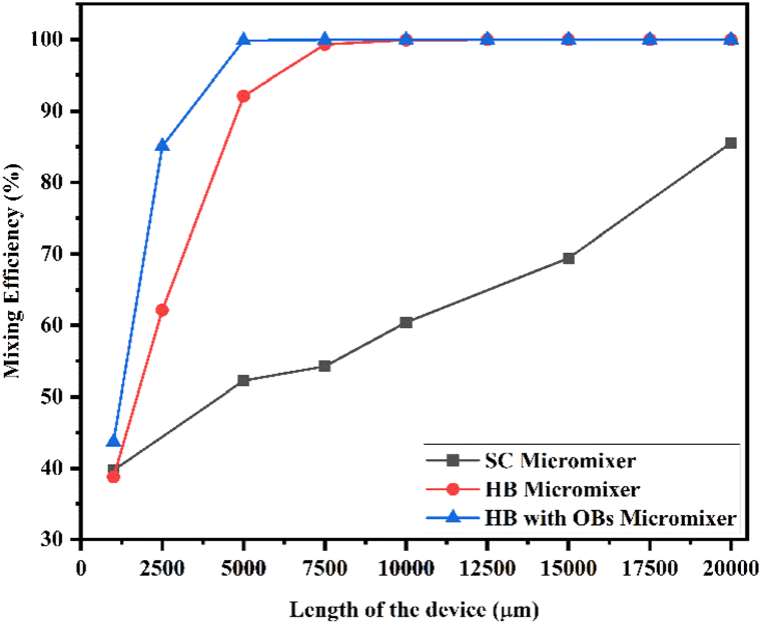
Mixing efficiency across the micromixer devices at 5 μL min^−1^ with a diffusion co-efficient of 15.3 × 10^−12^ m^2^ s^−1^.

Fig. 25 shows the mixing efficiency across the fluidic channel at different locations of 1000 μm, 2500 μm, 5000 μm, 7500 μm, 10 000 μm, 12 500 μm, 15 000 μm, 17 500 μm and 20 000 μm with a constant flow rate and a diffusion co-efficient of 15.3 × 10^−12^ m^2^ s^−1^. From Fig. 25, we are able to see three different micromixer devices: Y-shaped straight channel micromixer (SCM), Y-shaped herringbone serpentine shape micromixer (HBM) and Y-shaped herringbone serpentine shape micromixer with obstacles (HBM-OB). In this study, we can observe that the mixing efficiencies of the SCM device at the above-mentioned locations are 39.72%, 52.23%, 52.23%, 60.42%, 69.42% and 85.54%. The mixing efficiencies of the HBM device are 38.79%, 62.14%, 92.10%, 99.29%, 99.91%, 99.96%, 99.96%, 99.96% and 99.96%. Similarly, the mixing efficiencies of HBM-OB are 43.71%, 85.07%, 99.84%, 99.94%, 99.94%, 99.94%, 99.94%, 99.94% and 99.94%. When the mixing efficiency of all three types of micromixer device and mixing length are compared, the best mixing efficiency was achieved in the HBM-OB device due to the structural dimensions of the device and the obstacles. The obstacles induce fluid–fluid interaction, causing the better mixing to be achieved over a short duration compared with the other two types of micromixer device.

**Fig. 26:**
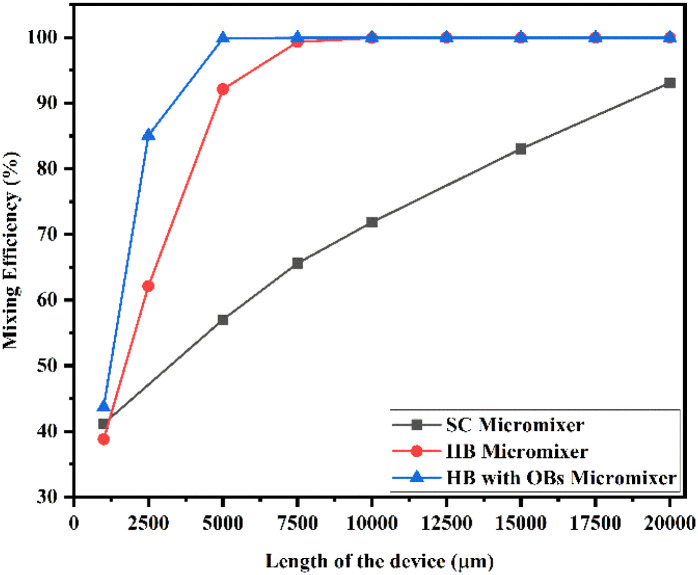
Mixing efficiency across the micromixer devices at 10 μL min^−1^ with a diffusion co-efficient of 15.3 × 10^−10^ m^2^ s^−1^.

Fig. 26 shows the mixing efficiency across the fluidic channel at different locations of 1000 μm, 2500 μm, 5000 μm, 7500 μm, 10 000 μm, 12 500 μm, 15 000 μm, 17 500 μm and 20 000 μm with a constant flow rate and a diffusion co-efficient of 15.3 × 10^−10^ m^2^ s^−1^. From Fig. 26, we are able to see three different micromixer devices: Y-shaped straight channel micromixer (SCM), Y-shaped herringbone serpentine shape micromixer (HBM) and Y-shaped herringbone serpentine shape micromixer with obstacles (HBM-OB). In this study, we can observe that the mixing efficiencies of the SCM device at the above-mentioned locations are 41.16%, 57.02%, 65.59%, 71.84%, 83.01% and 93.07%. The mixing efficiencies of the HBM device are 38.83%, 62.38%, 92.19%, 99.31%, 99.89%, 99.93%, 99.93%, 99.93% and 99.93%. Similarly, the mixing efficiencies of HBM-OB are 43.60%, 85.37%, 99.81%, 99.88%, 99.88%, 99.88%, 99.88%, 99.88% and 99.88%. When the mixing efficiency of all three types of micromixer device and mixing length are compared, the best mixing efficiency was achieved in the HBM-OB device due to the structural dimensions of the device and the obstacles.

**Fig. 27:**
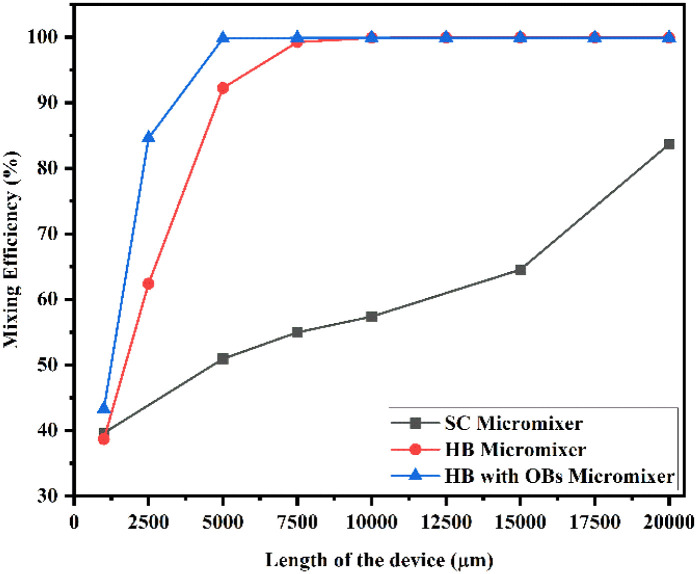
Mixing efficiency across the micromixer devices at 10 μL min^−1^ with a diffusion co-efficient of 15.3 × 10^−11^ m^2^ s^−1^.

Fig. 27 shows the mixing efficiency across the fluidic channel at different locations of 1000 μm, 2500 μm, 5000 μm, 7500 μm, 10 000 μm, 12 500 μm, 15 000 μm, 17 500 μm and 20 000 μm with a constant flow rate and a diffusion co-efficient of 15.3 × 10^−11^ m^2^ s^−1^. Fig. 27 illustrates three different types of micromixers: a Y-shaped straight channel micromixer (SCM), a Y-shaped herringbone serpentine shape micromixer (HBM) and a Y-shaped herringbone serpentine shape micromixer with obstacles (HBM-OB). The mixing efficiencies of the SCM device at the above-mentioned locations are 39.62%, 50.93%, 54.95%, 57.37%, 64.53% and 83.67%, respectively. The mixing efficiencies achieved by the HBM device are 38.67%, 62.39%, 92.24%, 99.25%, 99.90%, 99.95%, 99.95%, 99.95% and 99.95%, respectively. As for the mixing efficiencies of HBM-OB, 44,28%, 84.61%, 99.81%, 99.91%, 99.91%, 99.91%, 99.91%, 99.91%, 99.91%, 99.91%, 99.91%, 99.91% and 99.91% have been recorded. Due to the structural dimensions of the device and the obstacles present, the HBM-OB device showed the best mixing efficiency when comparing the mixing efficiency of the three types of micromixer. In comparison with the other two types of micromixer device, the obstacles induce fluid–fluid interaction, which results in improved mixing in a short period of time.

**Fig. 28:**
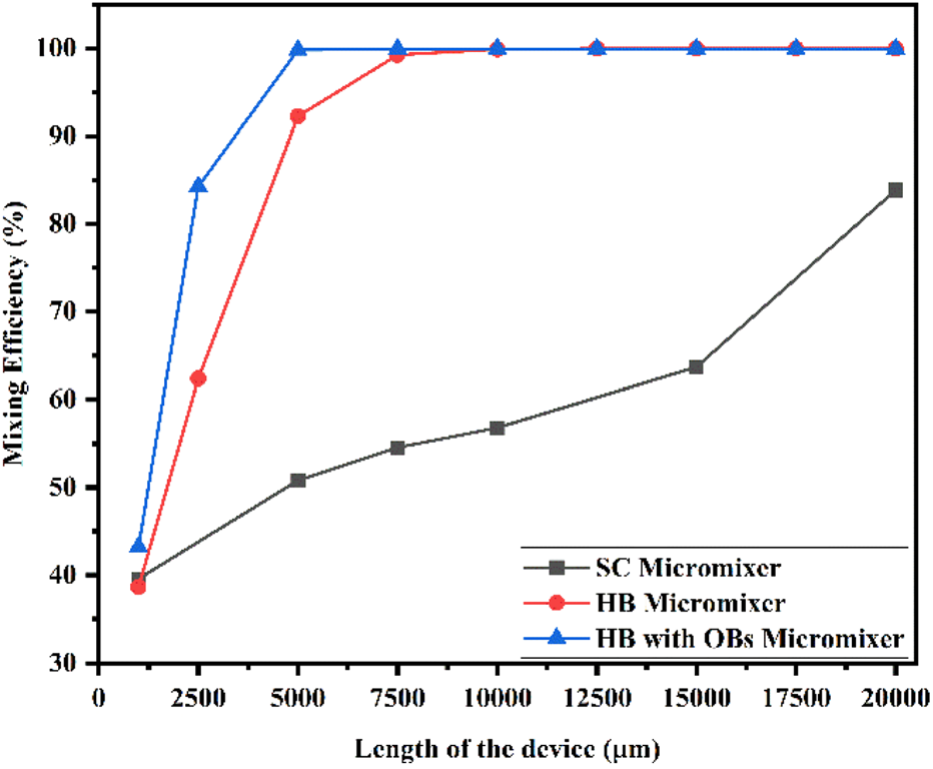
Mixing efficiency across the micromixer devices at 10 μL min^−1^ with a diffusion co-efficient of 15.3 × 10^−12^ m^2^ s^−1^.

Fig. 28 shows the mixing efficiency across the fluidic channel at different locations of 1000 μm, 2500 μm, 5000 μm, 7500 μm, 10 000 μm, 12 500 μm, 15 000 μm, 17 500 μm and 20 000 μm with a constant flow rate and a diffusion co-efficient of 15.3 × 10^−11^ m^2^ s^−1^.

From Fig. 28, we are able to see three different micromixer devices: Y-shaped straight channel micromixer (SCM), Y-shaped herringbone serpentine shape micromixer (HBM) and Y-shaped herringbone serpentine shape micromixer with obstacles (HBM-OB). In this study, we can observe that the mixing efficiencies of the SCM device at the above-mentioned locations are 39.58%, 50.81%, 54.56%, 56.81%, 63.70% and 83.89%. The mixing efficiencies of the HBM device are 38.63%, 62.38%, 92.27%, 99.25%, 99.91%, 99.97%, 99.97%, 99.97% and 99.97%. Similarly the mixing efficiencies of HBM-OB are 43.22%, 84.29%, 99.83%, 99.94%, 99.94%, 99.94%, 99.94%, 99.94% and 99.94%. When the mixing efficiency of all three types of micromixer device and mixing length are compared, the best mixing efficiency was achieved in the HBM-OB device due to the structural dimensions of the device and the obstacles.

Generally, a conventional mixer device requires a greater volume of samples and reagents and other existing micromixer devices also work in high flow rates to achieve complete mixing. This high flow rate will create a greater pressure drop (more than 10 kPa). This proposed and optimized micromixer device provides complete mixing in a shorter length with shorter timing and with a lower pressure drop.

**Fig. 29:**
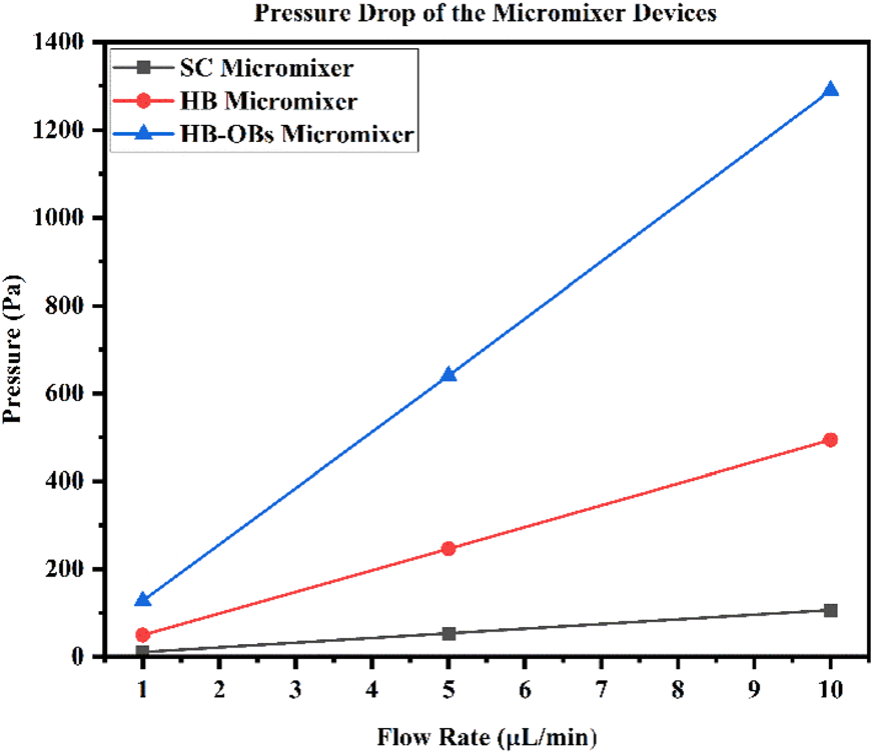
Pressure drops *vs.* flow rates in the micromixer devices.

Fig. 29 shows the pressure drop of micromixer devices with respect to three different flow rates of 1, 5 and 10 μL min^−1^. This figure shows the lowest pressure drop was observed when using an SCM device, such as 10.67, 53.302 and 106.74 Pa. The observed pressure drop levels in the HBM device are 49.37, 246.56 and 494.05 Pa and the pressure drop levels of HBM-OB are 128.01, 640.55 and 1289.7 Pa. We can arrange the pressure drop level of the device in the following order: HBM-OB > HBM > SCM device.

### 4.4 Grid independence verification

**Fig. 30:**
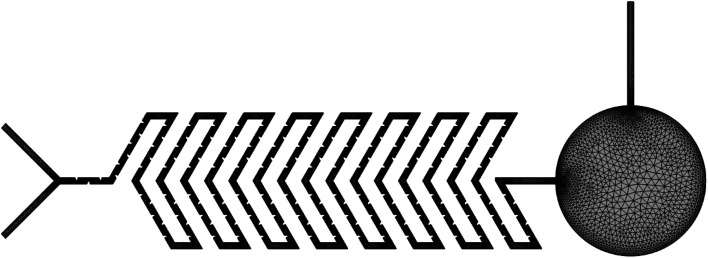
The mesh used for geometry.

The entire geometry is represented by an unstructured triangular mesh. Fig. 30 illustrates a typical mesh used in this study. A large number of flow gradients exist near the inlet, mixing zone, sensing zone, outlet, and close to the wall boundary in these regions. In order to capture the most detailed information possible, the mesh element size is refined in the regions of the obstacles. In addition to our simulations, we are also experimenting with mesh independence to determine the best mesh element size that will yield independent results. The average concentration at the channel outlet is given in Table 2 for three different mesh sizes for the main geometric design depicted in Fig. 3. Due to the negligible variation in concentration values from the third to the fourth row in Table 2, the mesh is determined based on the conditions found in the third row. Karthikeyan *et al.*^16^ provided numerical results that were compared to the simulation results of the current numerical method.

**Table 2:** Grid independence confirmation for the main geometry using Test case 19

Average concentration at 5000 μm	Number of elements
5.51616 mol m^−3^	40 948
5.49954 mol m^−3^	55 750
5.49793 mol m^−3^	65 516
5.49609 mol m^−3^	137 008

Table 3 shows a comparison of different types of passive micromixer device with different specifications.

**Table 3:** Comparison of different types of passive micromixer device

Type	Channel width	Channel height	Typical velocity	Mixing efficiency	Pressure drop	Reference
Y-shaped with obstacles	0.100 mm	0.100 mm	1.06 m s^−1^	0.05 MI	3200 Pa	12
Y-shaped with grooves	0.200 mm	0.100 mm	1–100 μL min^−1^	99.24%	0.01–1.1 × 10^5^ Pa	16
T-shaped with three different cross sectionals	0.3 mm	0.6 mm	1 × 10^−4^ m s^−1^	95%	0.5 × 10^5^ Pa	21
T-shaped with obstacles	0.150 mm	0.150 mm	0.1 ≤ Re ≤ 100	99.1%	18 135.8 Pa	22
Y-shaped with circular channel	0.200 mm	0.030 mm	1–6 μL min^−1^	97.08%	—	24
Y-shaped with ring	0.150 mm	0.155 mm	4–20 ml min^−1^	96–98%	—	26
T-shaped with 2 inlets and 2 outlets	0.150 mm	0.100 mm	0.09–0.5 mm s^−1^	0.45 mol m^−3^	2–6 Pa	27
T-shaped with obstacles	0.100 mm	0.100 mm	0.006 m s^−1^	0.500 mol m^−3^	—	29
Y-shaped serpentine channel	0.100 mm	0.100 mm	9–75 Re	100%	23 Pa	34
T-shaped with obstacles	0.300 mm	0.100 mm	0.001–0.1 and 40–45 Re	90–100%	—	35
Y-shaped serpentine shape	0.300 mm	0.300 mm	0.28 to 30 Re	0.9 to1.0 MI	7500 Pa	39
T-shaped with different obstacles	0.2 mm	0.2 mm	0.04 m s^−1^	81.2%	2600 Pa	40
3D T-shaped	0.10 mm	0.050 mm	25 to 250 Re	54%	29 kPa	41
Y-shaped with square and circle	0.3 mm	0.2 mm	100 Re	99.9%	65 MPa	42
Tesla micromixer	0.200 mm	0.200 mm	1–100 μL min^−1^	0.45 mol m^−3^	52.868 Pa	43
Y-shaped herringbone serpentine channel micromixer with obstacles	0.200 mm	0.100 mm	1, 5, 10 μL min^−1^	100% at 5000 μm	128 Pa	Present work*

### 4.5 Time domain analysis across the device

**Fig. 31:**
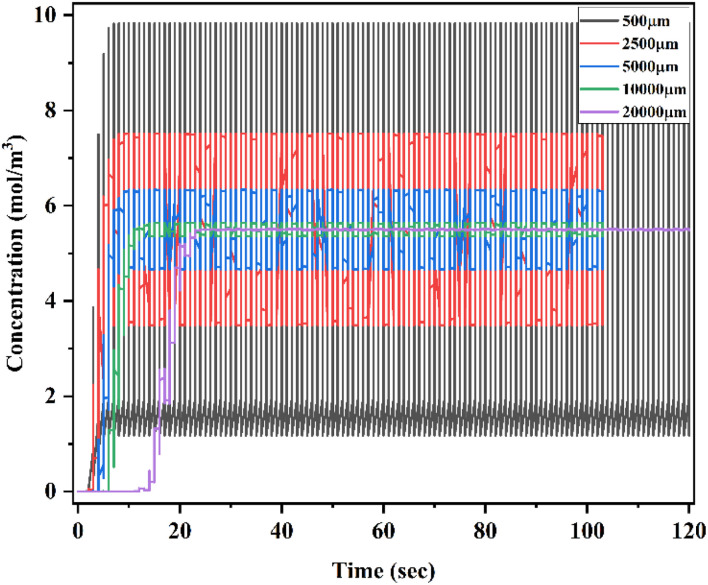
Time domain study of Y-shaped straight channel micromixer at 1 μL min^−1^ with a diffusion co-efficient of 15.3 × 10^−10^ m^2^ s^−1^.

Fig. 31 shows a time domain study of the SCM device at a low flow rate of 1 μL min^−1^ with a diffusion co-efficient of 15.3 × 10^−10^ m^2^ s^−1^ (TC1). The *x*-axis denotes the time (seconds) and the *y*-axis denotes the concentration (mol m^−3^) at different locations of the device of 500 μm, 2500 μm, 5000 μm, 10 000 μm and 20000 μm. This time domain study shows the change in concentration level at different locations and timings. This study was carried out for 120 seconds from inlet to outlet. At the initial stage of 500 μm, there was a wide concentration level between 1 and 10 mol m^−3^ and there was not complete mixing; when it was measured at 2500 μm, the concentration range was between 3.80 and 7.40 mol m^−3^. Similarly, at 5000 μm, there was a broad concentration level between 4.8 and 6.1 mol m^−3^ and there was not complete mixing; when it was measured at 10 000 μm, the concentration range was between 5 and 6 mol m^−3^. Finally, at 20 000 μm, the concentration level was almost narrow: 5.5 mol m^−3^ at 24 seconds.

**Fig. 32:**
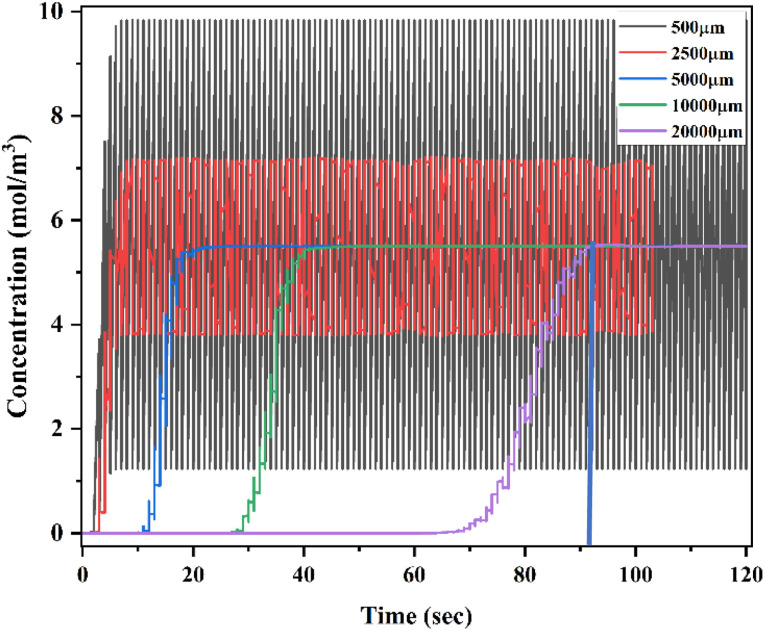
Time domain study of Y-shaped herringbone serpentine channel micromixer at 1 μL min^−1^ with a diffusion co-efficient of 15.3 × 10^−10^ m^2^ s^−1^.

Fig. 32 shows a time domain study of the HBM device at a low flow rate of 1 μL min^−1^ with a diffusion co-efficient of 15.3 × 10^−10^ m^2^ s^−1^ (TC10). At different locations of the device, of 500 μm, 2500 μm, 5000 μm, 10 000 μm, and 20 000 μm, the *x*-axis represents time in seconds and the *y*-axis represents concentration in mol m^−3^.

An analysis of concentration levels over time at different locations and timings is presented in this study. During this experiment, the inlet and outlet were monitored for 120 seconds. Initially, the concentration level was wider at 500 μm, ranging from 1–10 mol m^−3^, while at 2500 μm, the concentration range was between 3.93 and 7.10 mol m^−3^. A similar concentration level was observed at 5000 μm when it was measured at 21 seconds, and it was 5.5 moles m^−3^ when it was measured at 10 000 μm and 20 000 μm at 41 seconds and 91 seconds, respectively.

**Fig. 33:**
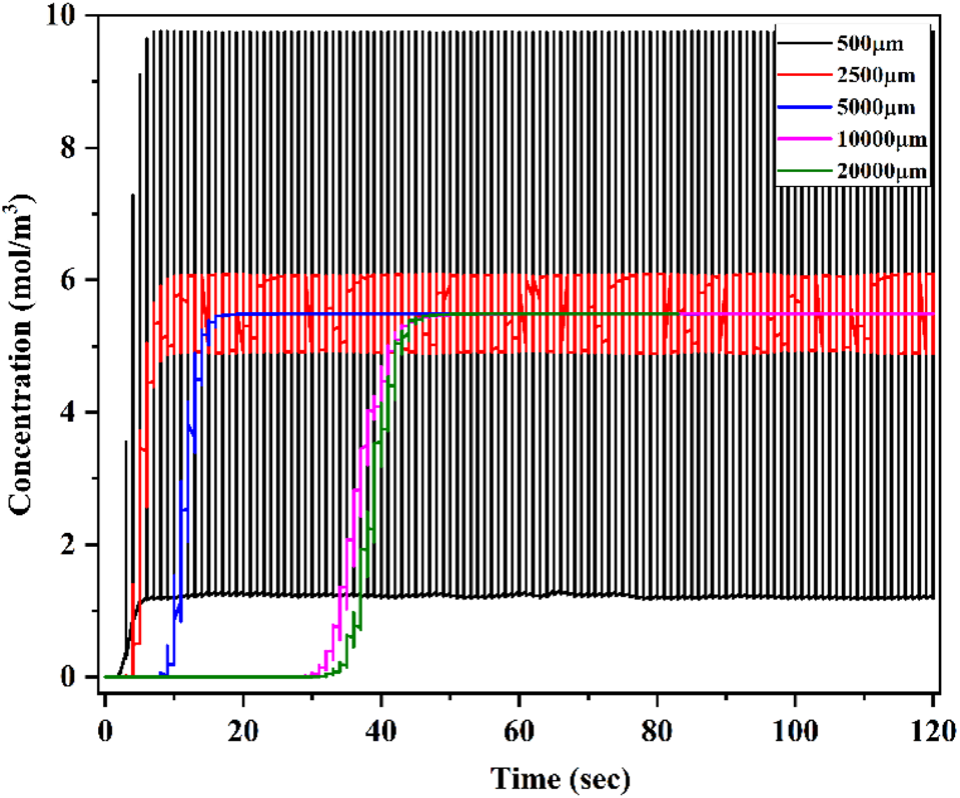
Time domain study of Y-shaped herringbone serpentine channel micromixer with obstacles at 1 μL min^−1^ with a diffusion co-efficient of 15.3 × 10^−10^ m^2^ s^−1^.

Fig. 33 shows a time domain study of the HBM-OB device at a low flow rate of 1 μL min^−1^ with a diffusion co-efficient of 15.3 × 10^−10^ m^2^ s^−1^ (TC19). The *x*-axis denotes the time (seconds) and the *y*-axis denotes the concentration (mol m^−3^) at different locations of the device of 500 μm, 2500 μm, 5000 μm, 10 000 μm and 20 000 μm. This time domain study shows the change in concentration level at different locations and timings. This study was carried out for 120 seconds from inlet to outlet. At the initial stage of 500 μm, there is a wide concentration level between 1 and 10 mol m^−3^ and there was not complete mixing; when it was measured at 2500 μm, the concentration range was between 4.8 and 6 mol m^−3^. Similarly, at 5000 μm, the concentration level was almost narrow at 5.5 mol m^−3^ at 16 seconds, and at this stage the fluid was completely and well mixed; when it was measured at 10 000 μm and 20 000 μm, the concentration level was almost saturated at 5.5 mol m^−3^ at 40 and 48 seconds. When comparing Test cases 1, 10 and 19, Test case 19 shows the best mixing efficiency within a short duration compared with the other test cases.

## 5 Conclusion

In this study, we have taken three different passive micromixer devices, SCM, HBM and HBM-OB, that have two inlets, a sensing zone and one outlet for mixing two fluids with three different diffusivities and three different flow rates, designed and analyzed using COMSOL Multiphysics software. In order to study the mixing performance of two different concentrations of inlet fluid (10 mol m^−3^ and 1 mol m^−3^) when the fluids are mixed completely the concentration will reach 5.5 mol m^−3^ and this will be considered as the point of complete mixing. Achieving this mixed point will be different from device to device, based on the structural dimensions as well as the input parameters.

A fuzzy logic program was used to classify the data obtained from the analyses, and optimization procedures were performed on the data. During the optimization process, the parameters were changed to obtain the data. Changes in input parameters were applied to the same design in order to obtain output data.

As a result of the analysis and optimization processes, the optimum input parameters that should be applied to the HBM-OB micromixer device in order to achieve complete mixing from the inlet fluids with flow rates of 1, 5, and 10 μL min^−1^ and a wide range of diffusivity 15.3 e^−10^, 15.3 e^−11^, and 15.3 e^−12^ m^2^ s^−1^ were determined. If the input parameters are applied to the microfluidic device in these value ranges, it is understood that the pressure in the output channel is in the range of 128–1289 Pa and complete mixing was achieved within a short length of less than 5000 μm and a short time of 10 seconds due to the structural dimensions of the device as well as the input parameters (TC19). The proposed HBM-OB micromixer device is most suitable for low-diffusivity fluids and its applications such as biosensing, blood plasma analysis, blood cell analysis and heavy metal ion sensing.

## Conflicts of interest

There are no conflicts to declare.

## Supplementary Material
